# Exploring the diversity and antimicrobial potential of actinomycetes isolated from different environments in Saudi Arabia: a systematic review

**DOI:** 10.3389/fmicb.2025.1568899

**Published:** 2025-03-26

**Authors:** Noof Refat Helmi

**Affiliations:** Department of Clinical Microbiology and Immunology, Faculty of Medicine, King Abdulaziz University, Jeddah, Saudi Arabia

**Keywords:** actinomycetes, *Streptomyces*, antimicrobial activity, antimicrobial resistance, extreme environment, bioactive compound, Saudi Arabia

## Abstract

The increasing prevalence of antimicrobial resistance (AMR) presents a significant global health challenge, underscoring the urgent need for novel antimicrobial agents. Actinomycetes, particularly *Streptomyces* species, are well known for synthesizing bioactive compounds with antibacterial, antifungal, and antiviral properties. This review explores the diversity and antimicrobial potential of actinomycetes from Saudi Arabia’s unique ecosystems, including terrestrial (soil, rhizosphere), aquatic (marine, freshwater), extreme (deserts, caves, hot springs, mountains, and mangroves), and other unique environments. The adaptation of these microorganisms to harsh environmental conditions has driven the evolution of unique strains with enhanced biosynthetic capacities. Several studies have demonstrated their antimicrobial efficacy against multidrug-resistant pathogens, including methicillin-resistant *Staphylococcus aureus* (MRSA), extended-spectrum beta-lactamase (ESBL)-producing Enterobacteriaceae, *Pseudomonas aeruginosa*, and *Candida albicans*. However, challenges in actinomycete research persist, including difficulties in culturing rare strains, limited genomic characterization, and high production costs. Recent advancements, such as genome mining, metagenomics, AI-driven bioinformatics, and CRISPR-based gene activation, offer promising avenues for unlocking novel antimicrobial compounds. Additionally, synthetic biology, advanced fermentation technologies, and nanotechnology-based drug delivery systems are enhancing the industrial scalability of actinomycete-derived antibiotics. Beyond antimicrobials, actinomycete-derived compounds show potential applications in oncology, immunotherapy, and agriculture. Alternative therapeutic strategies, including quorum sensing inhibitors, phage therapy, and combination therapies, are being explored to combat AMR. Cutting-edge analytical techniques, such as mass spectrometry, liquid chromatography, and nuclear magnetic resonance spectroscopy (NMR), are essential for structural elucidation and mechanism characterization of new bioactive compounds. To harness Saudi Arabia’s microbial biodiversity effectively, interdisciplinary collaborations between microbiologists, biotechnologists, and pharmaceutical industries are crucial. Sustainable bioprospecting and advanced bioprocessing strategies will facilitate the translation of actinomycete-derived bioactive compounds into clinically viable therapeutics. Expanding research efforts into underexplored Saudi ecosystems can lead to groundbreaking discoveries in antibiotic development and beyond.

## 1 Introduction

Antimicrobial resistance (AMR) has emerged as a critical global health challenge, significantly impacting infection management and clinical outcomes. The increasing prevalence of resistant microbial strains has led to heightened morbidity and mortality rates, particularly among vulnerable populations, including children, the elderly, and immunocompromised individuals. The World Health Organization (WHO) estimates that by 2050, AMR could lead to 10 million deaths annually, emphasizing the urgent need for novel antimicrobial agents (Alanis, 2005; Mancuso et al., 2021; Naghavi et al., 2024).

Multidrug-resistant (MDR) pathogens, including methicillin-resistant *Staphylococcus aureus* (MRSA) and vancomycin-resistant *Staphylococcus aureus* (VRSA), have emerged as critical concerns, alongside vancomycin-resistant *Enterococcus* spp. (VRE) and penicillin-resistant *Streptococcus pneumoniae* (PRSP), which further complicate treatment options. Extended-spectrum beta-lactamase (ESBL)- producing *Escherichia coli* (*E. coli*) and *Klebsiella pneumoniae* (*K. pneumoniae*) have been increasingly reported, alongside carbapenem-resistant *Enterobacteriaceae* (CRE), which exhibit resistance to last-line *β*-lactam antibiotics. Additionally, non-fermenting Gram-negative bacteria, including multidrug-resistant *Pseudomonas aeruginosa* (*P. aeruginosa*) and *Acinetobacter baumannii* (*A. baumannii*), have emerged as significant nosocomial pathogens with limited therapeutic options. This trend has been extensively documented in recent literature (Zowawi, 2016; Alotaibi, 2019; Thabit et al., 2023; Kiran et al., 2024). Moreover, the rise of multidrug-resistant *Mycobacterium tuberculosis* (MDR-TB) further exacerbates the global burden of antimicrobial resistance (Yezli and Memish, 2012).

Given the alarming rise in MDR pathogens and the diminishing efficacy of existing antibiotics, the search for novel antimicrobial compounds has become imperative. However, the decline in pharmaceutical investment in antibiotic discovery poses a major challenge to addressing this crisis. The extended time required for toxicological and clinical investigations, high costs, and lower profitability compared to other drug categories have significantly slowed the development of new antibiotics (Alenazi et al., 2023). As a result, researchers have increasingly turned to natural microbial biodiversity as a promising source of novel antimicrobial agents with potent bioactivity and minimal toxicity.

Among these microbial taxa, actinomycetes—particularly the genus *Streptomyces*—have historically been prolific producers of bioactive secondary metabolites, accounting for approximately 75% of clinically relevant antibiotics. While extensive research has focused on soil-derived actinomycetes, species isolated from extreme and underexplored environments have demonstrated exceptional potential for the biosynthesis of novel antimicrobial compounds (Aly et al., 2019; Al-Shaibani et al., 2021; Jagannathan et al., 2021; Alaidaroos, 2022). However, these niche ecosystems remain largely untapped, warranting further investigation.

In this context, exploring geographically unique and environmentally challenging habitats offers an opportunity to discover novel actinomycetes with distinct antimicrobial properties. Saudi Arabia’s diverse ecosystems—including deserts, coastal regions, caves, and mountainous terrains—constitute an underexplored reservoir of actinomycetes with significant antimicrobial potential (Al-Ghamdi et al., 2021; Almuhayawi et al., 2021; Albasri et al., 2024). The extreme environmental conditions of these habitats impose selective pressures that drive the evolution of unique biosynthetic gene clusters (BGCs), which may serve as promising sources for antibiotic discovery and biotechnological applications (Adegboye and Babalola, 2012). Despite their potential, actinomycetes from these distinct ecological niches remain largely uncharacterized.

This review aims to systematically assess the antimicrobial activities of actinomycetes from Saudi Arabia’s environments, elucidate novel bioactive compounds, and explore their applications in addressing antibiotic resistance. By bridging this knowledge gap, we seek to contribute to the identification of new therapeutic agents and advance our understanding of microbial diversity in challenging ecological niches.

## 2 Methodology

### 2.1 Literature search strategy

This systematic review was conducted following the Preferred Reporting Items for Systematic Reviews and Meta-Analyses (PRISMA) guidelines to ensure transparency and rigor. A comprehensive literature search was performed across multiple scientific databases, including PubMed, Scopus, Web of Science, ScienceDirect, and Google Scholar, to identify relevant studies on actinomycetes in Saudi Arabia. Additionally, reference lists of selected articles were manually screened for additional sources. Relevant scientific books were also consulted to provide historical and foundational insights.

The search terms used included:

- “Actinomycetes in Saudi Arabia”
- “Antimicrobial properties of actinomycetes”
- “Antimicrobial resistance”
- “Extremophilic actinomycetes”
- “Bioactive compounds from actinomycetes”

The literature search covered studies published up to 2024 to ensure the inclusion of the most recent and relevant findings.

### 2.2 Eligibility criteria

The inclusion and exclusion criteria were established to ensure the selection of high-quality and relevant studies.

Inclusion Criteria:

1. Peer-reviewed publications focusing on actinomycetes from Saudi Arabia.
2. Research on the antimicrobial activity and bioactive compound production of these isolates.
3. Experimental studies involving isolation, characterization, and bioactivity screening of actinomycetes.
4. Studies of antimicrobial resistance in Saudi Arabia to provide context for the significance of new antimicrobial agents.
5. Comparative studies that include actinomycetes from multiple environments (e.g., terrestrial, aquatic, extreme habitats).
6. Articles published in English.

Exclusion Criteria:

1. Non-peer-reviewed sources, including conference abstracts and opinion articles.
2. Studies lacking clear and comprehensive data presentation.
3. Studies irrelevant to the core focus of this review.
4. Articles with limited accessibility or those not available through major scientific databases.

### 2.3 Study selection process

The study selection process followed the PRISMA flowchart approach:

1. Identification: All retrieved records from the database searches were compiled, and duplicates were removed.
2. Screening: Titles and abstracts were reviewed to exclude irrelevant studies.
3. Eligibility Assessment: Full-text articles were assessed against the inclusion and exclusion criteria.
4. Inclusion: Eligible studies were included in the final qualitative and quantitative synthesis.

### 2.4 Data extraction and analysis

A standardized data extraction sheet was used to collect information from the selected studies. Extracted data included:

- Study details (author, year of publication)
- Sampling locations
- Identified actinomycete strains
- Antimicrobial activity (spectrum and potency)
- Bioactive compounds detected
- Methods used for antimicrobial activity assessment

A comparative analysis was conducted to identify trends in actinomycete diversity, antimicrobial properties, and their distribution across various environments in Saudi Arabia. The findings were synthesized to highlight the ecological and pharmacological significance of these microorganisms.

### 2.5 Risk of bias assessment

To ensure the reliability and validity of the included studies, a risk of bias assessment was performed using appropriate tools based on study design. Studies were evaluated for methodological quality, completeness of data reporting, and potential conflicts of interest. The results of the risk of bias assessment were documented to account for variations in study quality.

By adopting the PRISMA framework, this systematic review aims to provide a comprehensive and transparent synthesis of the available literature on actinomycetes in Saudi Arabia.

## 3 Actinomycetes: biological characteristics, bioactive secondary metabolites, and antibiotic biosynthesis

Actinomycetes are a diverse group of Gram-positive bacteria classified under the phylum *Actinomycetota* within the domain *Bacteria*.^1^ These microorganisms are characterized by a high guanine-cytosine (G + C) content in their genomic DNA, which contributes to their genetic stability and adaptability. The term “actinomycetes” was derived from the Greek words “aktis” (a ray) and “mykes” (fungus), reflecting their filamentous morphology (Chaudhary et al., 2013; Siro and Pipite, 2024). Historically, actinomycete were considered transitional form between bacteria and fungi due to their mycelial growth pattern. However, they are definitively classified as prokaryotes based on their cellular organization, possessing nuclei without a membrane-bound structure and a peptidoglycan-based cell wall. Their structural and physiological diversity is extensive, encompassing filamentous and non-filamentous forms, motile and non-motile variants, as well as aerobic and anaerobic species. Furthermore, they exhibit variability in their reproductive strategies, forming either spores or non-spore-bearing structures. A distinguishing characteristic of actinomycetes is their ability to develop both aerial and substrate mycelia, along with structures such as sporangia and conidial chains, which enhance their survival and adaptation in extreme environments. Their colonies typically exhibit a powdery texture and adhere firmly to the agar surface, a feature that aids in their identification and differentiation from other bacterial taxa (Goodfellow et al., 2012). Additionally, actinomycetes demonstrate intrinsic resistance to various antibacterial agents, a trait that has contributed to their significance in biotechnology and antibiotic production.

**Figure 1:**
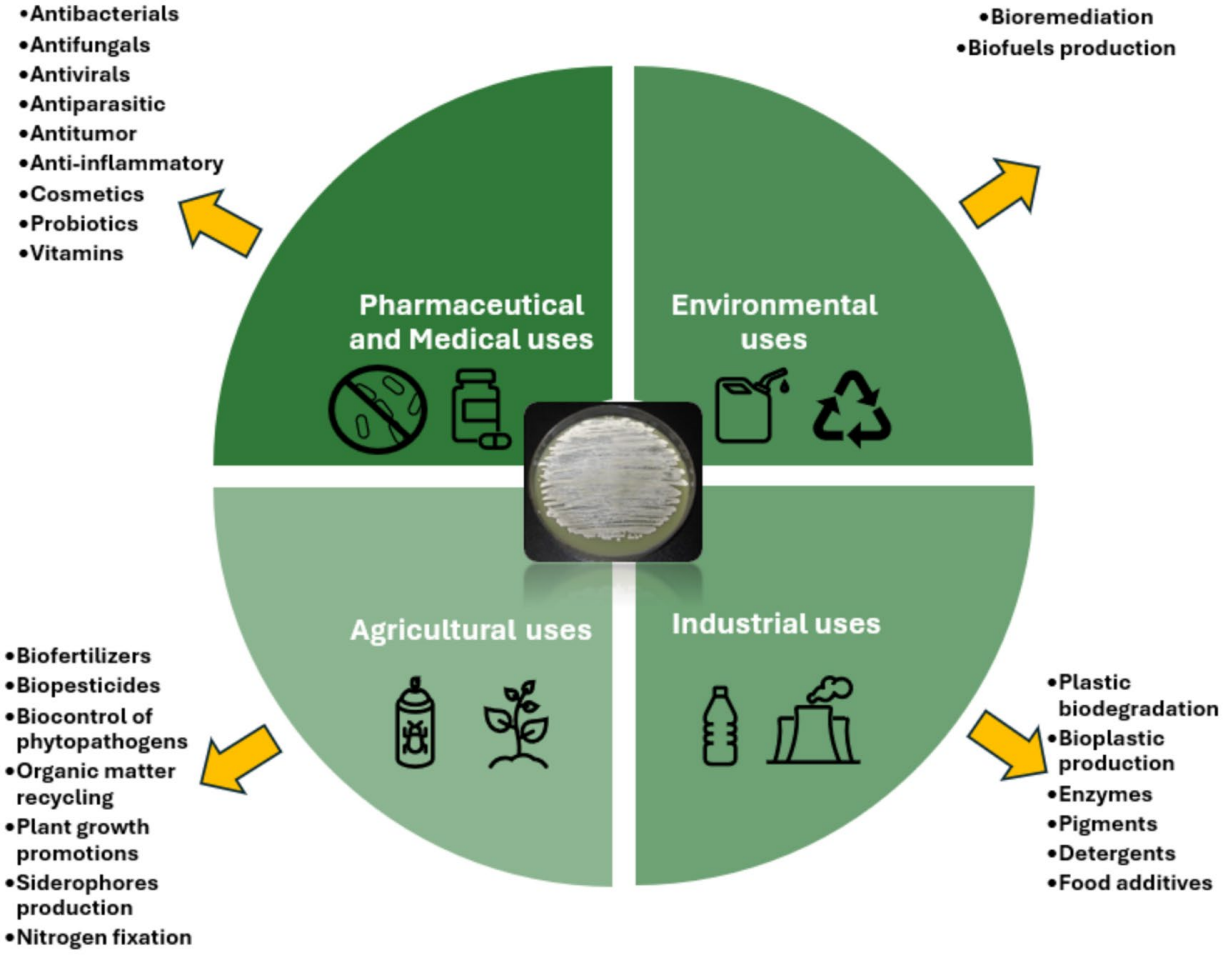
Actinomycetes applications and products.

Actinomycetes are recognized for their ability to produce bioactive secondary metabolites that exhibit a diverse array of biological effects, including antibacterial, antifungal, antiviral, antiparasitic, anticancer, and immunosuppressant effects, as well as enzymes and other bioactive compounds (Bérdy, 2005; Kekuda et al., 2010; Chaudhary et al., 2013; Jagannathan et al., 2021). These compounds have wide-ranging applications in environmental, agricultural, industrial, pharmaceutical, and clinical fields (Figure 1). Their biosynthetic potential is largely attributed to BGCs encoding polyketide synthases (PKSs), nonribosomal peptide synthetases (NRPSs), and other enzymes involved in the production of bioactive compounds (Adegboye and Babalola, 2012). Recent advancements in comparative genomics have significantly enhanced the discovery and optimization of antibiotic biosynthetic pathways, providing new insights into their metabolic potential. Moreover, metagenomics and synthetic biology approaches have emerged as powerful tools for uncovering novel secondary metabolites and improving their yield through genetic modifications (Mitousis et al., 2020; van Bergeijk et al., 2020; Jagannathan et al., 2021).

Among the approximately 22,000 known microbial secondary metabolites, actinomycetes account for 70–75% of these bioactive compounds, with fungi contributing around 20%, *Bacillus* species 7%, and other bacterial taxa collectively representing only 1–3%. In terms of antibiotic production, actinomycetes are responsible for approximately 50–55% of all clinically established antibiotics. Within this group, the genus *Streptomyces* contributes nearly 75% of these compounds, while the remaining 25% are produced by non-*Streptomyces* species, commonly referred to as “rare actinomycetes” (Bawazir et al., 2019; Dimri et al., 2020; Ngamcharungchit et al., 2023).

Rare actinomycetes include genera distinct from *Streptomyces* or those infrequently isolated under standard laboratory conditions. These genera include *Actinomadura, Actinoplanes, Amycolatopsis, Actinokineospora, Acrocarpospora*, *Actinosynnema*, *Actinopolyspora*, *Actinoalloteichus*, *Catenuloplanes*, *Cryptosporangium*, *Dermabacter, Dactylosporangium*, *Frankia, Kytococcus, Kibdelosporangium*, *Kineosporia*, *Kutzneria*, *Microbacterium, Micromonospora*, *Microbiospora*, *Microtetraspora*, *Micropolyspora, Nocardia*, *Nocardiopsis*, *Nonomuraea*, *Planomonospora*, *Planobispora*, *Pseudonocardia*, *Rhodococcus, Saccharomonospora*, *Saccharopolyspora, Saccharothrix*, *Streptosporangium*, *Streptoverticillium*, *Spirilliplanes*, *Salinispora*, *Thermoactinomyces, Thermomonospora*, *Thermopolyspora, Thermobifida*, *Verrucosispora,* and *Virgosporangium* (Lazzarini et al., 2000; Khanna et al., 2011; Tiwari and Gupta, 2012; Tiwari and Gupta, 2013; Hug et al., 2018). These taxa are increasingly recognized as valuable sources of novel bioactive compounds, particularly considering the growing challenge of antimicrobial resistance.

Actinomycetes have historically been the primary source of clinically important antimicrobial agents, including *β*-lactams (e.g., cephamycin), polyketides, glycopeptides (e.g., vancomycin), ansamycins (e.g., rifamycin), macrolides (e.g., erythromycin), tetracycline, aminoglycosides, chloramphenicol, quinolones, sulfonamides, polyenes, polyethers, nucleosides, lipopeptides (e.g., daptomycin), peptides, and numerous other bioactive molecules (Baltz, 2007; Mahajan and Balachandran, 2012; Alharbi, 2016; Barka et al., 2016; De Simeis and Serra, 2021). Among actinomycetes, *Streptomyces* species have been particularly prolific in producing antimycobacterial agents, with many compounds exhibiting potent activity against *Mycobacterium* species. Notable examples include caprazamycin B, sansanmycins, urdamycinone E, urdamycinone G, dehydroxyaquayamycin, streptcytosine A, chrysomycin A, and dinactin (Alharbi, 2016; Al-Dhabi et al., 2020a). These metabolites underscore the potential of *Streptomyces* and other actinomycetes as invaluable reservoirs for the discovery of novel antimicrobial agents.

## 4 Ecological diversity and antimicrobial potential of actinomycetes in Saudi Arabia: insights from terrestrial, aquatic, and extremophilic environments

**Figure 2:**
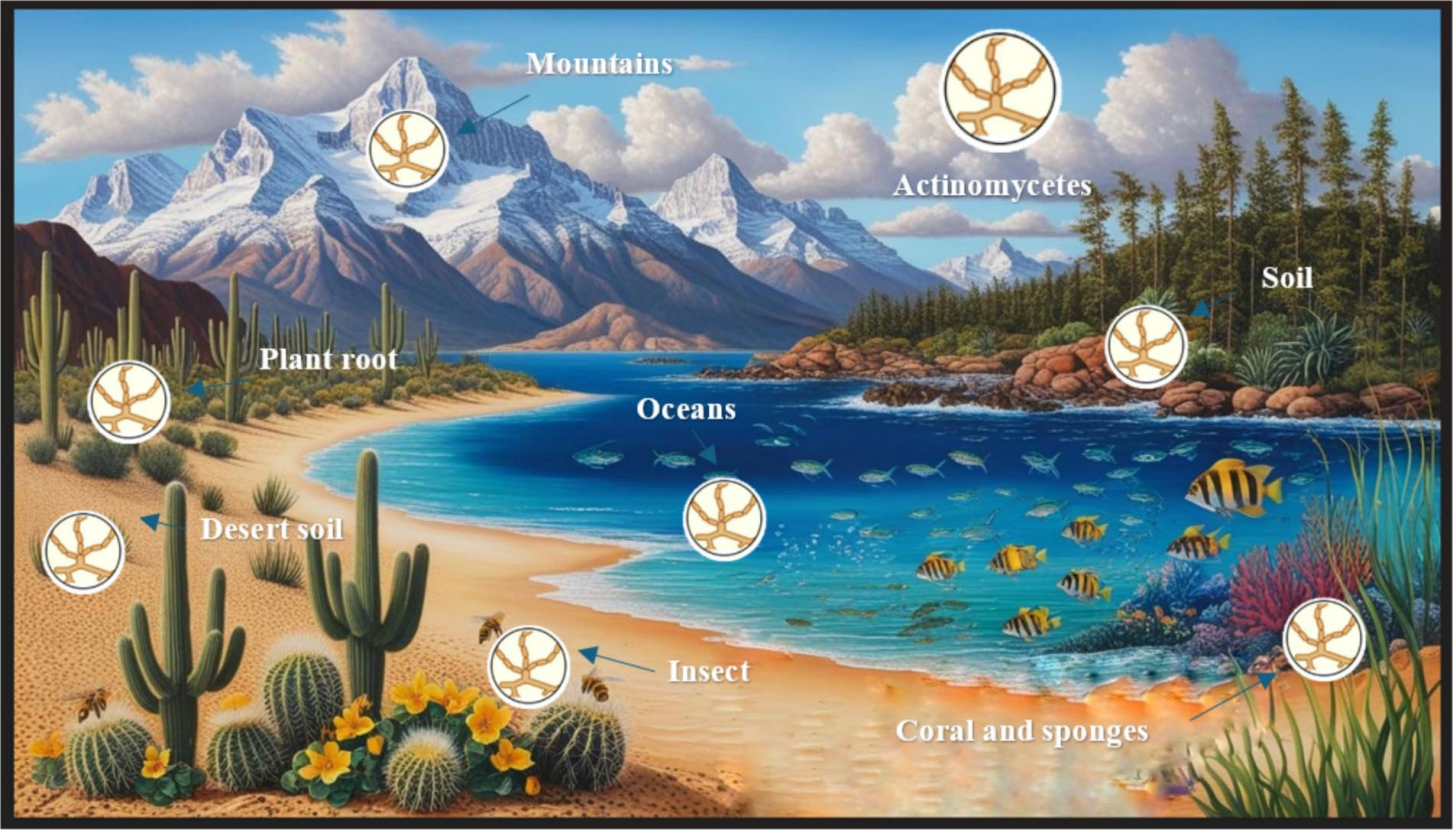
Diverse habitats of actinomycetes.

Actinomycetes are ubiquitous microorganisms present in several ecosystems, including terrestrial, aquatic, and extremophilic environments (Figure 2). They live as free-living saprophytic bacteria. Some isolates can live inside the tissues of plants, insects, or aquatic animals. They adapt well to a range of conditions, including usual and harsh habitats characterized by elevated or reduced temperatures, intense radiation, acidic or alkaline pH levels, high salinity, and settings with restricted moisture or nutrients. These organisms have important applications in biotechnology and medicine due to their ability to produce a variety of bioactive compounds with antimicrobial activity (Zenova et al., 2011; Meenakshi et al., 2024).

### 4.1 Terrestrial environments

#### 4.1.1 Soil

Actinomycetes are primarily free-living microorganisms located in diverse habitats. They are also well known as soil saprophytes and are responsible for the distinctive earthy scent of freshly plowed soil due to the synthesis of geosmin (Sharma et al., 2014). Among soil actinomycetes, *Streptomyces* is the most dominant genus. However, the diversity and bioactivity of actinomycetes are significantly influenced by environmental factors such as geographical location, soil temperature, type, pH, organic matter content, agricultural activities, aeration, nutrient availability, moisture content, and surrounding vegetation (Arifuzzaman et al., 2010). They are especially prevalent in slightly alkaline organic rich soils, where they synthesize diverse secondary metabolites essential for medical and agricultural applications. Their ecological roles extend beyond nutrient cycling to include nitrogen fixation (e.g., *Frankia* sp.), solubilization and immobilization of nutrients, siderophore production, and biocontrol activity against soil-borne pathogens (Dilip et al., 2013).

Numerous studies across different regions have underscored the significant antimicrobial potential of soil-derived actinomycetes. Studies from India (Pandey et al., 2011; Patel et al., 2014) have identified diverse soil-derived actinomycetes strains from agricultural soils, riversides and grassland areas, often linked to high organic content and moderate climate conditions producing antimicrobial compounds against multidrug-resistant pathogens such as *Staphylococcus aureus* (*S. aureus*), *E. coli*, and *P. aeruginosa*, and antifungal properties. Similar findings have been reported from Egypt (Elbendary et al., 2018; Sebak et al., 2021), identified actinomycetes that exhibit inhibitory effects against a wide array of pathogens, including *S. aureus*, MRSA, *Bacillus cereus* (*B. cereus*), *E. coli*, *K. pneumoniae*, *P. aeruginosa*, *Salmonella typhi* (*S. typhi*), *Candida albicans* (*C. albicans*), *Aspergillus niger* (*A. niger*), and *Aspergillus flavus* (*A. flavus*). Recent work by Atallah et al. (2023a) identified *Streptomyces rochei* (*S. rochei*), which exhibited significant antimicrobial activities against *Bacillus subtilis* (*B. subtilis*), *Salmonella enteritidis* (*S. enteritidis*), and *P. aeruginosa*. Awad et al. (2024) reported similar findings, with actinomycetes exhibiting antibacterial effects against *S. aureus*, MRSA, and *P. aeruginosa*, as well as antifungal activity, particularly against *Aspergillus fumigatus* (*A. fumigatus*) and *Fusarium solani* (*F. solani*).

Unlike temperate and tropical regions, Saudi Arabia soils exhibit environmental conditions, including high temperatures, aridity, and variable salinity, favoring the survival of stress-tolerant actinomycetes with specialized secondary metabolites (Alharbi et al., 2012; Al-Kadeeb et al., 2012; Mohamed et al., 2013b). Similar ecological trends have been observed in actinomycetes from the Atacama Desert, where extremophilic strains have demonstrated unique biosynthetic capabilities, producing compounds distinct from those of mesophilic strains (Okoro et al., 2009; Rateb et al., 2011).

Numerous investigations in Saudi Arabia have proven the significant antimicrobial potential of soil-derived actinomycetes (Table 1). The discovery of *Streptomyces flavogriseus* from agricultural soils in Riyadh and Qassim (Al-Kahtani, 2016) and *S. rochei* from Al-Kharj (Muharram et al., 2013) showed broad-spectrum antimicrobial activity against Gram-positive bacteria such as *S. aureus* and *B. subtilis*, as well as Gram-negative bacteria like *E. coli* and antifungal activity against *C. albicans* and *A. niger*. More recently, Ibnouf et al. (2022) documented additional antimicrobial-producing actinomycetes from Al-Kharj, isolating bioactive compounds with diverse medical uses, including antibacterial, antifungal, anti-inflammatory, antioxidant, and anti-cancer characteristics. Some of these compounds also serve as essential ingredients in cosmetics. Additionally, Ababutain et al. (2012a, 2012b) identified a *Streptomyces* strain from Dammam synthesizing lincomycin-like antibiotics, effective against both Gram-positive and Gram-negative bacteria, as well as insects. Abid et al. (2016) recovered *Streptomyces* strains from Jazan with activity against bacteria like *B. subtilis*, *S. aureus*, *P. aeruginosa*, *E. coli*, and *Shigella sonnei* (*S. sonnei*) with inhibition zones ranging from 3.25 to 26.875 mm in diameter. In terms of antifungal activity, these strains were effective against *Fusarium moniliforme*, *Helminthosporium oryzae*, *A. flavus*, *Aspergillus japonicus*, and *Fusarium verticillioides* with an inhibition zone ranging from 13.3 to 40 mm. Similar findings were reported by Sheik et al. (2017) in Ad-Dawadmi and Jastaniah et al. (2019b) in Jeddah, where *Streptomyces* spp. showed antibacterial efficacy against *E. coli, K. pneumoniae, P. aeruginosa, S. aureus,* MRSA, *and Enterococcus faecalis* (*E. faecalis*). These investigations underscore the pharmaceutical potential of Saudi soil-derived actinomycetes, especially *Streptomyces*, as valuable sources of innovative antimicrobial compounds. The diversity of these isolates and their extensive efficacy against antibiotic-resistant bacteria and harmful fungi highlight their significance in combating the global issue of antimicrobial resistance.

**Table 1:** Diversity and antimicrobial activity of soil-derived actinomycetes in Saudi Arabia.

Specimen type	Isolation site/ Study area	Isolated strains	Antimicrobial activity	Methods used to assess activity	Test microorganisms	Bioactive/chemical compound(s)	Reference
Soil	Riyadh	*-Streptomyces albus* *-Streptomyces diastaticus* *-Streptomyces atroolivaceous* *-Streptomyces violaceus* *-Streptomyces exfoliate* *-Streptomyces niveus*	Antibacterial Antifungal	Cork-borer (Plug) method	*-Escherichia coli* *-Klebsiella pneumoniae* *-Pseudomonas aeruginosa* *-Staphylococcus aureus* *-Bacillus subtilis* *-Streptococcus pyogenes* *-Saccharomyces cerevisiae* *-Candida albicans* *-Aspergillus flavus* *-Fusarium solani*	NA	Al-Kadeeb et al. (2012)
Sandy/ Clay Soil	Dammam-Riyadh Road Dammam Khobar Dhahran Jubail Unaizah	Actinomycetes isolates; *Streptomyces* sp. MS-266 Dm4	Antibacterial Antifungal	Agar well diffusion methods	*-Pseudomonas aeruginosa* *-Escherichia coli* *-Bacillus cereus* *-Bacillus subtilis* *-Candida albicans* *-Aspergillus flavus* *-Aspergillus niger*	Active substance belonging to lincomycin antibiotic	Ababutain et al. (2012a, 2012b)
Soil	Al-Kharj	*-Streptomyces sampsonii* *-Streptomyces albidoflavus* *-Streptomyces roche*	Antibacterial Antifungal	Cross-streak, Cork-borer and Agar well methods	*-Staphylococcus aureus* *-Bacillus subtilis* *-Escherichia coli* *-Candida albicans* *-Aspergillus niger*	Crude extract	Muharram et al. (2013)
Soil	Jazan	*-Streptomyces* strains: JS3, JS4, JS6, JD7, JA8 and JA10	Antibacterial Antifungal	Agar well diffusion method	*-Bacillus subtilis* *-Staphylococcus aureus* *-Pseudomonas aeruginosa* *-Escherichia coli* *-Shigella sonnei* *-Fusarium* spp. *-Aspergillus flavus* *-Aspergillus japonicus*	Crude extract	Abid et al. (2016)
Agricultural soil	Riyadh and Qassim	*Streptomyces* species; *Streptomyces flavogriseus*	Antibacterial	Cross streak and disc diffusion methods	*-Staphylococcus aureus* *-Bacillus subtilis* *-Escherichia coli* *-Candida albicans*	Crude extract	Al-Kahtani et al. (2016)
Soil	Ad-Dawadmi	*Streptomyces* species; DOM1, DOM3, DP3, DP4.	Antibacterial	Cross streak and Agar well diffusion methods	*-Escherichia coli* *-Pseudomonas aeruginosa* *-Staphylococcus aureus* *-Enterococcus*	Crude extract	Sheik et al. (2017)
Garden soil	Jeddah	*Streptomyces* SD5	Antibacterial	Agar disc and Agar well diffusion methods	*-Escherichia coli* *-Klebsiella pneumoniae* *-Pseudomonas aeruginosa* *-Enterococcus faecalis* -MRSA	Crude extract	Jastaniah et al. (2019b)
Sandy soil	Al-Kharj	Actinomycetes isolates	Antibacterial Antifungal	Cross streak and Agar well diffusion methods	*-Staphylococcus aureus* *-Bacillus subtilis* *-Escherichia coli* *-Klebsiella pneumoniae* *-Pseudomonas aeruginosa* *-Proteus vulgaris* *-Salmonella typhimurium* *-Aspergillus niger* *-Candida albicans*	1. Hexadecane, 2,6,11,15- Tetramethyl 2. Octacosane 3. Dodecanoic Acid, 1,2,3- Propanetriyl ester 4. Hexatriacontane 5. Heptacosane 6. Eicosyl Acetate 7. Tritetracontane 8. Tetracosane, 2,6,10,15,19,23-Hexamethyl 9. Myristic Acid vinyl ester 10. Tetratetracontane	Ibnouf et al. (2022)

NA: Not Available MRSA: Methicillin-resistant *Staphylococcus aureus*.

#### 4.1.2 Rhizosphere soil

The rhizosphere—the soil microzone surrounding plant roots—is a crucial ecological niche where actinomycetes play a significant role in nutrient cycling, organic matter decomposition, and suppression of soil-borne pathogens. Through complex interactions with plant roots, rhizosphere actinomycetes enhance soil fertility, plant health, and resistance against pathogenic microbes (Adegboye and Babalola, 2012). This symbiotic interaction is essential for fostering sustainable agriculture by diminishing the necessity for chemical-based fertilizers and pesticides. In addition to their agricultural benefits, many rhizosphere-derived actinomycetes, especially from genera such as *Streptomyces*, *Micromonospora*, *Microbispora*, *Nocardiopsis*, *Rhodococcus*, and *Nocardia*, synthesize bioactive compounds with antibacterial, antifungal, antiviral, and anticancer properties (Matsumoto and Takahashi, 2017; Singh and Dubey, 2018; Alzahrani et al., 2022).

Extensive global studies have demonstrated the role of rhizosphere actinomycetes in plant growth promotion and phytopathogen control (Gohain et al., 2015; Refaat et al., 2017; Elsayed et al., 2020). *Streptomyces* sp. CAH29 from the rhizosphere has yielded bioactive molecules such as tetrangomycin, which exhibits antibacterial and antifungal activity against *S. aureus*, *Streptococcus pyogenes* (*S. pyogenes*), MRSA, and *C. albicans*, with inhibition zones of 14, 10, 12, and 8 mm, respectively (Özakin et al., 2016). Similarly, picolinamycin, isolated from an Indian *Streptomyces* strain, demonstrated significant antibacterial activity against *S. aureus* with minimum inhibitory concentration (MIC) of 0.04–5.12 μg/mL, and moderate anti-mycobacterial effects at higher concentrations (MIC: 10.24 μg/mL) (Maiti et al., 2020).

In contrast, research in Saudi Arabia has primarily focused on the diversity and characterization of rhizosphere actinomycetes, with limited studies investigating their role as biocontrol agents. However, emerging evidence suggests that these microorganisms could serve as promising candidates for agricultural applications. Ahmed et al. (2021) obtained actinomycetes strains from the rhizosphere of *Satureja hortensis* in Jouf area of Saudi Arabia. The phenolic content of these strains exhibited significant antioxidant, antibacterial, and antiprotozoal action against *Trypanosoma cruzi* (*T. cruzi*). Similarly, *Streptomyces spororaveus* RDS28, recovered from rhizosphere soils in Riyadh, exhibited broad-spectrum antifungal activity (Al-Askar et al., 2011). Further, *Streptomyces griseorubens* E44G demonstrated potent antagonism against *Fusarium oxysporum* (*F. oxysporum*) (Al-Askar et al., 2014). More recently, *Streptomyces abyssalis* SHR13, isolated from rhizosphere soils in Jeddah and Yanbu Al-Nakheel, exhibited inhibitory activity against *F. oxysporum, A. niger, K. pneumoniae,* and *P. aeruginosa* (Khallaf et al., 2023).

Additional studies in Saudi Arabia have expanded the scope of rhizosphere actinomycete research. *Streptomyces ramulosus*, identified from the catnip rhizosphere in Al-Madinah Al-Munawwarah, demonstrated antibacterial action against both Gram-negative and Gram-positive pathogens (Abo-Shadi et al., 2010). Furthermore, *Streptomyces flavogriseus* (KF235416), isolated from *Acacia tortilis* rhizosphere soil, exhibited potent antimicrobial activity against a range of pathogens, MRSA, VRE, *A. baumannii*, *S. pyogenes*, *E. coli*, *Salmonella typhimurium* (*S. typhimurium*), and *Shigella* spp., in addition to fungi such as *Aspergillus* spp. and *C. albicans* (Al-Garni et al., 2014). These findings suggest that Saudi Arabia harbors untapped biocontrol agents for agricultural use. Table 2 shows the diversity and antimicrobial activity of actinomycetes from rhizosphere soil in Saudi Arabia.

**Table 2:** Diversity and antimicrobial activity of rhizosphere soil -derived actinomycetes in Saudi Arabia.

Specimen type	Isolation site/ Study area	Isolated strains	Antimicrobial activity	Methods used to assess activity	Test microorganisms	Bioactive/chemical compound (s)	Reference
Rhizosphere soil (Tomato, cucumber, alfalfa and onion)	Riyadh	*-Streptomyces spororaveus* RDS28	Antifungal	Cork-borer and disc diffusion methods	*-Rhizoctonia solani* *-Fusarium* spp. *-Alternaria alternata* -*Botrytis cinerea*	Crude extract	Al-Askar et al. (2011)
Rhizosphere soil	Al-Ahsaa, Al Jouf, Al-Kharj, Al-Madenah, Al-Qaseem, Al-Qatif, Al-Quwayiyah, Al-Sulayyil, Al-Ta’if, Hail, Jeddah, Gazan, Makkah, Najran, Riyadh, Shagra, Tabuk Wadi Al-Dawasir	Actinomycetes Isolates; *Streptomyces griseorubens* E44G	Antifungal Antibacterial	Disc diffusion method	*-Fusarium solani* *-Fusarium oxysporum* *-Macrophomina phaseolina* *-Alternaria radicina* *-Rhizoctonia solani* *-Sclerotium rolfsii* *-Nigrospora oryzae* *-Phoma destructive -Penicillium notatum* *-Aspergillus niger* *-Staphylococcus aureus* *-Streptococcus pneumoniae* *-Pseudomonas aeruginosa* *-Escherichia coli* *-Bacillus subtilis*	Culture filtrate	Al-Askar et al. (2014)
Rhizosphere soil (Corn, datepalm, catnip, sunflower, balessan, nabk-tree, basil)	AlMadinah Al-Munawwarah, Al-Aziziah and Al-Owinah	Actinomycetes Isolates; *Streptomyces ramulosus* A-MM-24	Antibacterial	Agar well, Cork borer and Streak methods	*-Escherichia coli* *-Klebsiella* spp. *-Pseudomonas* spp. *-Proteus* spp. *-Citrobacter* spp. *-Acinetobacter* spp. -MRSA -CONS	Culture filtrate	Abo-Shadi et al. (2010)
Rhizosphere soil (*Acacia tortilis, Citrus paradisi, Morus*)	Al-Madinah Al-Munawwarah	Actinomycetes isolates; D8 *Streptomyces flavogriseus*, D9, D27, D39, D 42.	Antibacterial Antifungal	Cork-borer and well diffusion methods	*-Staphylococcus aureus* *-Escherichia coli* *-Streptococcus pyogenes* -MRSA -VRE *-Acinetobacter baumannii* *-Salmonella typhimurium* *-Shigella* sp. *-Candida albicans* *-Aspergillus niger* *-Aspergillus flavus*	Culture filtrate	Al-Garni et al. (2014)
Rhizosphere soil	Jeddah, Yanbu Al-Nakheel	*Streptomyces abyssalis* (SHR13)	Antibacterial Antifungal	Agar well diffusion method	*-Staphylococcus aureus* *-Enterobacter aerogenes* *-Acinetobacter baumannii* *-Escherichia coli* *-Klebsiella pneumoniae* *-Pseudomonas aeruginosa* *-Fusarium oxysporum* *-Fusarium redolense* *-Curvularia khuzestanica* *-Rhizoctonia solani* *-Aspergillus niger* *-Candida albicans*	Culture filtrate	Khallaf et al. (2023)
Rhizosphere soil (*Satureja hortensis*)	Al Jouf	*Streptomyces species* (Ac1 Ac2 Ac3 Ac4 Ac5 Ac6 Ac7 Ac8 Ac9)	Antibacterial Antiprotozoal	Different methods	*-Streptococcus* sp. *-Escherichia coli* *-Trypanosoma cruzi*	Flavonoids and Phenolic acids	Ahmed et al. (2021)

MRSA: Methicillin-resistant *Staphylococcus aureus* CONS: Coagulase negative *Staphylococcus* VRE: Vancomycin-resistant *Enterococcus* spp.

While global studies have led to significant antibiotic discoveries, and actinomycete-based biofertilizers and biopesticides have been developed and commercialized, Saudi research remains in a developmental phase, with great potential for future biotechnological applications. Addressing underexplored environments- rhizosphere of native Saudi plants, such as *Acacia* and *Zygophyllum* species-could unlock novel actinomycetes with specialized bioactive metabolites, contributing to global efforts in drug discovery and sustainable agriculture.

### 4.2 Aquatic environments

Actinomycetes from aquatic habitats have historically received less attention compared to their terrestrial counterparts. However, these microorganisms, known for their prolific production of bioactive compounds, have been extensively isolated from both freshwater and marine environments (Aly et al., 2019; Al-Shaibani et al., 2021) (see Table 3).

**Table 3:** Diversity and antimicrobial activity of marine-derived actinobacteria in Saudi Arabia.

Specimen type	Isolation site/ Study area	Isolated strains	Antimicrobial activity	Methods used to assess activity	Test microorganisms	Bioactive/ Chemical compound (s)	Reference
Soil, water sediments, marine water, wastewater, marine shrimps & well water.	Jeddah	*Streptomyces* spp. (*Streptomyces* sp. BM21)	Antifungal Anticandidal	Disc diffusion and Agar well diffusion methods	*-Candida albicans* *-Candida tropicals* *-Alterneria solani* *-Aspergillus flavus* *-Fusarium oxysporum* *-Penicillium italicum -Rhizopus nigricans*	Protein	Aly et al. (2011)
Sea water, Sea sediment soil & soil	Jeddah Taif	*Streptomyces* spp.	Antibacterial Antifungal	Disc diffusion method	*-Salmonella* sp. *-Staphylococcus* sp. *-Escherichia coli* *-Bacillus subtilis* *-Aspergillus* sp. *-Rhizoctonia* sp. *-Fusarium* sp.	Culture filtrate	Mohamed et al. (2013a)
Sea water, Shrimp shell, Well water, Sewage, Sand of beach & Sand	Jeddah	Actinomycetes isolates (D73)	Antifungal	Agar well diffusion method	*-Staphylococcus aureus* *-Escherichia coli* *-Salmonella typhimurium* *-Aspergillus niger* *-Aspergillus flavus*	Culture filtrate	Al-Garni et al. (2014)
Marine sponges	Red Sea (Thuwal, Fsar Reef)	*-Micrococcus* spp. *Micromonospora* sp. *Salinispora* spp. *Kocuria* spp. *Nocardia* sp. *Rhodococcus* spp. *Mycobacterium* sp. *Saccharomonospora* spp. *Rothia* sp. *Dietzia* sp.	Antibacterial Antifungal Antiparasitic Antiviral	Disk diffusion method (Antibacterial and Antifungal) The IC_50_ values (Anti-Trypanosomal) Protease Assay Kit (SensoLyte 440) (Antiviral)	*-Bacillus* sp. *-Escherichia coli* *-Fusarium* sp. *-Trypanosoma brucei* *-Leishmania major* -West Nile Virus	Crude extracts	Abdelmohsen et al., 2014
Marine sediments	Jeddah	*Streptomyces* spp. *Streptomyces mutabilis* ML51	Antibacterial	Agar plug and gar well-diffusion methods	*-Staphylococcus aureus* -MRSA *-Enterococcus faecalis* *-Acinetobacter baumannii* *-Klebsiella oxytoca* *-Escherichia coli*	Crude extract	Bahamdain (2020)
Marine sediments	Arabian ocean (Dammam and Al-Khobar)	*Streptomyces* sp. Al-Dhabi-89	Antibacterial	Well diffusion and broth micro-dilution methods	*-Escherichia coli* *-Klebsiella pneumoniae* *-Pseudomonas aeruginosa* *-Acinetobacter baumannii* *-Staphylococcus aureus* *-Proteus mirabilis* *-Enterococcus faecium*	Silver nanoparticles	Al-Dhabi et al. (2018)
Soil, sediment, water from the seashore, sponges & crabs	Arabia Gulf regions	*Streptomyces* sp. Al-Dhabi-90	Antibacterial	Perpendicular cross streak, well diffusion and broth micro-dilution methods	-*Enterococcus faecalis* *-Bacillus subtilis* *-Staphylococcus aureus* -*Staphylococcus epidermidis* *-Escherichia coli* *-Klebsiella pneumoniae* *-Pseudomonas aeruginosa* -*Proteus mirabilis* *-Acinetobacter baumannii* -VRE	3-methylpyridazine, *n*-hexadecanoic acid, indazol-4-one, and 3a-methyl-6-(4-methylphenyl) sul as the major compounds	Al-Dhabi et al. (2019b)
Soil sediment from the seashore	Dammam, Saihat, AL Khobar & Dhahran	*Streptomyces* sp. Al-Dhabi-97	Antibacterial	Streaked perpendicular, disc diffusion and broth micro-dilution methods	-*Bacillus subtilis* -*Enterococcus faecalis* -*Staphylococcus epidermidis* *-Staphylococcus aureus* *-Pseudomonas aeruginosa* *-Klebsiella pneumoniae* *-Escherichia coli* -*Salmonella paratyphi*	1-phenanthrenemethanol, phthalic acid, di(2-propylpentyl) ester, benzenebutanoic acid, podocarp-7-en-3-one, indole-3-carboxaldehyde as the major compounds	Al-Dhabi et al. (2020b)
Marine sediment, Marine soil, Marine water, Marine rock powder, Marine fishes & marine decayed plants	Jazan	*Streptomyces* sp. Al-Dhabi-100	Antibacterial Antitubercular	Cross streak perpendicular, cup plate diffusion, disc diffusion and broth micro dilution methods Luciferase Reporter Phage (LRP) assays (Antitubercular)	*-Enterococcus faecalis* *-Bacillus subtilis* -*Staphylococcus aureus* -*Staphylococcus epidermidis* *-Klebsiella pneumoniae* *-Escherichia coli* *-Pseudomonas aeruginosa* -*Mycobacterium tuberculosis*	1-(2,6-Dimethyl-4-propoxyphenyl)propan-1-one and ethyl 2-propylphenyl ester as the major compounds	Al-Dhabi et al. (2020a)

MRSA: Methicillin-resistant *Staphylococcus aureus* IC_50_: Half maximal inhibitory concentration VRE: Vancomycin-resistant *Enterococcus* sp.

#### 4.2.1 Marine

Marine ecosystems, encompassing over two-thirds of the surface of the earth, are distinguished by significant variations in the temperature, salinity, nutrient availability, oxygen levels, and pressure. These factors contribute to the extensive microbial diversity of marine habitats and influence the genetic adaptation of marine actinomycetes, leading to the biosynthesis of distinct secondary metabolites that differ from those of their terrestrial counterparts (Subramani and Sipkema, 2019; Mothana et al., 2022; Chakraborty et al., 2023). The successful isolation of rare marine actinomycetes requires a comprehensive understanding and precise control of key environmental parameters, including pH, culture temperature, oxygen levels, and nutritional requirements. To optimize the cultivation of these microorganisms, growth media should closely mimic the osmotic conditions of seawater. Notably, sodium ions (Na^+^) represent a critical component in culture media, as they are essential for the growth and physiological activity of marine actinomycetes (Subramani and Sipkema, 2019).

While earlier studies questioned whether marine actinomycetes were merely terrestrial contaminants, subsequent research has confirmed the existence of indigenous marine actinomycetes that require seawater for optimal growth (Fenical and Jensen, 2006). Marine actinomycetes belong to a diverse array of genera, including *Actinoplanes*, *Actinomycetospora*, *Agrococcus*, *Aeromicrobium*, *Arsenicicoccus*, *Arthrobacter, Brevibacterium*, *Citricoccus*, *Dietzia*, *Geodermatophilus*, *Janibacter*, *Kocuria*, *Microbacterium*, *Microlunatus*, *Micromonospora*, *Nocardia*, *Nocardioides*, *Nocardiopsis*, *Rhodococcus*, *Salinispora*, *Sciscionella*, *Serinicoccus*, *Saccharopolyspora*, *Salinibacterium*, *Streptomyces*, and *Thermoactinomyces* (Subramani and Aalbersberg, 2013; Alharbi, 2016; Bawazir et al., 2019; Subramani and Sipkema, 2019). Notably, marine-derived actinomycetes produce a wide range of novel bioactive compounds, including pacificanones, salinipyrones, rifamycins, arenimycin, cyanosporaside A, saliniketals A and B, marinomycin A–D, abyssomicin-C, and others, which exhibit antimicrobial, antifungal, and anticancer properties, underscoring their pharmaceutical potential (Subramani and Aalbersberg, 2012; Subramani and Sipkema, 2019).

Marine sediments are recognized as the primary reservoir of actinomycetes; however, they have also been isolated from diverse marine environments, including seawater, sand, rocks, and marine flora and fauna such as sponges (Abdelmohsen et al., 2014), corals (Mahmoud and Kalendar, 2016), seagrass (Siro and Pipite, 2024), fish (Jagannathan et al., 2021), mollusks (Dilip et al., 2013), shrimps (Ali et al., 2020), and algae (El-Gendy et al., 2008).

Several studies have highlighted the significant antimicrobial efficacy of marine actinomycetes against MDR bacterial and fungal pathogens, demonstrating their potential as a valuable source of novel antimicrobial agents (Valli et al., 2012; Hegazy et al., 2023). For instance, actinomycetes isolated from the Bay of Bengal, predominantly *Streptomyces* species, displayed potent antimicrobial activity against *S. aureus*, *S. typhi*, *B. cereus*, *E. coli*, *K. pneumoniae*, and phytopathogenic fungi (Kalyani et al., 2012; Mohanraj and Sekar, 2013). Similarly, *Streptomyces griseorubens* and *S. rochei*, recovered from the black sand shores of Egypt, exhibited strong antimicrobial activity against MDR pathogens such as *B. subtilis, S. enteritidis,* and *P. aeruginosa* (Atallah et al., 2023b). Additionally, Ribeiro et al. (2020) isolated 52 actinomycetes from marine sediments along the Portuguese coast, primarily from the *Micromonospora* genus. Six *Streptomyces* strains exhibited antifungal activity against *C. albicans*, with MIC values ranging from 3.9 to 125 μg/mL.

Saudi Arabia’s marine environments, including the Red Sea and the Arabian Gulf, along with various inland freshwater bodies (wadis, oases, and artificial lakes), present extreme conditions that influence microbial diversity and metabolic adaptations. The Red Sea and Arabian Gulf, in particular, harbor halotolerant actinomycetes that have adapted to high salinity and fluctuating temperatures. For example, *Actinopolyspora saudiensis* sp. nov., a halophilic actinomycete isolated from hypersaline soil in Jeddah, exhibits exceptional salt tolerance, thriving at NaCl concentrations of up to 30%, underscoring its potential in biotechnological uses in saline conditions (Kiki and Al Turk, 2013). Several studies have demonstrated the antimicrobial potential of marine actinomycetes from these environments (Nadeem et al., 2015; Abdelfattah et al., 2016; El Samak et al., 2018; Hamed et al., 2019). Marine-derived *Salinispora* and *Micromonospora* strains have been identified as prolific producers of bioactive metabolites with potent antifungal and antibacterial properties, presenting promising leads for antibiotic development (Mahmoud and Kalendar, 2016; Alqahtani et al., 2022; Mothana et al., 2022).

Additionally, actinomycetes isolated from the Red Sea have exhibited remarkable antimicrobial activity. Aly et al. (2011) recovered 90 actinomycete strains from soil, marine sediments, seawater, wastewater, marine shrimp, and well water in Jeddah. Among these, 10 isolates displayed strong inhibitory effects against *C. albicans*, with *Streptomyces* sp. BM21 producing a protein that disrupted fungal cell wall regeneration. Similarly, Mohamed et al. (2013a) identified 18 *Streptomyces*-like isolates from Red Sea sediments, demonstrating substantial antimicrobial activity. Further investigations by Al-Garni et al. (2014) resulted in the isolation of 81 actinomycete strains from Jeddah and Al-Madinah Al-Munawwarah, many of which exhibited broad-spectrum antimicrobial properties. Notably, *Streptomyces* sp. Al-Dhabi-100 from Jazan demonstrated potent antibacterial activity against *E. faecalis*, *B. subtilis*, and *S. aureus*, along with anti-tubercular and antioxidant properties (Al-Dhabi et al., 2020a). Marine sponge-associated actinomycetes from the Red Sea have also proven to be a valuable source of bioactive compounds. Abdelmohsen et al. (2014) isolated 48 actinomycetes strains, with extracts from 9 strains showing significant antibacterial, antifungal, antiparasitic, and antiviral activities. Notably, two strains, identified as *Salinispora* spp., exhibited broad-spectrum inhibition against multiple pathogenic microorganisms. Similarly, Abdelmohsen et al. (2010) also explored the marine environment, isolating 90 actinomycetes strains from sponges collected near Ras Mohamed, Egypt, and Rovinj, Croatia. Additionally, Shamikh et al. (2020) focused on actinomycetes associated with marine sponges collected from Hurghada, Egypt. This study identified novel species from the genera *Micromonospora*, *Nocardia*, and *Gordonia*, which showed activity against *S. aureus*, *E. faecalis*, *C. albicans*, and *Trypanosoma brucei*.

The Arabian Gulf has similarly yielded actinomycetes with promising biomedical applications. *Streptomyces* sp. Al-Dhabi-89, isolated from Dammam, demonstrated potent antibacterial activity against MDR pathogens such as *E. coli*, *K. pneumoniae*, *P. aeruginosa*, *A. baumannii, Proteus mirabilis* (*P. mirabilis*), multidrug-resistant *S. aureus* and *Enterococcus faecium* (*E. faecium*), with MIC values ranging from 7.81 to 62.5 μg/mL (Al-Dhabi et al., 2018). Likewise, *Streptomyces* sp. Al-Dhabi-90 exhibited antibacterial activity against vancomycin-resistant *Enterococcus faecium* and ESBL-producing *E. coli* and *P. aeruginosa* (Al-Dhabi et al., 2019b). Moreover, *Streptomyces* sp. Al-Dhabi-97, isolated from Saudi Arabian seashores, displayed broad-spectrum antimicrobial activity, with MIC values ranging from 62.5 to 500 μg/mL (Al-Dhabi et al., 2020b).

These findings highlight the vast biotechnological potential of marine actinomycetes from Saudi Arabia’s aquatic ecosystems, reinforcing their role as promising sources for novel antimicrobial agents and pharmaceutical applications.

#### 4.2.2 Freshwater

While marine ecosystems, with their high salinity and nutrient limitations, provide an environment for extremophilic actinomycetes, Saudi Arabia’s freshwater ecosystems—including seasonal wadis, oases, artificial lakes, dams, and underground aquifers— remain largely unexplored despite their potential to harbor diverse microbial communities. The seasonal fluctuations in water availability and salinity within these freshwater environments may contribute to microbial diversity, including actinomycetes with significant biotechnological potential.

Although marine actinomycetes have been extensively studied, freshwater environments have also been recognized as reservoirs of bioactive actinomycetes. Several studies have reported the isolation of actinomycetes from freshwater sources with potent antimicrobial activities. For instance, *Streptomyces viridiviolaceus*, isolated from Lake Bardawil in Egypt, exhibited broad-spectrum antibacterial activity (Rabeh et al., 2007). Similarly, genera such as *Saccharopolyspora* and *Actinosynnema*, isolated from freshwater sources in South Africa, demonstrated antimicrobial potential against both Gram-positive and Gram-negative bacteria (Sibanda et al., 2010). Further evidence from the Nile River in Egypt (Aly and El-Sabbagh, 2004), Lake Fetzara in Algeria (Benhadj et al., 2019), Lonar Lake in India (Kharat et al., 2009), and Lake Tana in Ethiopia (Gebreyohannes et al., 2013) has underscored the presence of actinomycetes producing bioactive compounds with antibacterial, antifungal, and anticancer properties.

Given the ecological dynamics of Saudi Arabia’s freshwater ecosystems, comprehensive multi-seasonal sampling and genomic studies are necessary to elucidate actinomycete diversity under varying hydrological conditions. Such investigations would facilitate the identification of novel bioactive compounds, advancing our understanding of their potential applications in medicine and biotechnology.

### 4.3 Extremophilic environments

Actinomycetes are known to occur in typical conditions, as well as in extreme and underexplored environments, including deserts (Li et al., 2019), caves (Farda et al., 2022), hot springs (AlSediy et al., 2022), eruptions of volcanoes (Ruwandeepika et al., 2022), mangroves (Law et al., 2019), mountainous areas (Albasri et al., 2024), thermal industrial wastes (Alaidaroos, 2022), Arctic and Antarctic regions (Augustine et al., 2012; Encheva-Malinova et al., 2014). Their ability to survive in such conditions is attributed to their physiological and metabolic flexibility, including the formation of resistant spores (arthrospores) and the production of secondary metabolites that facilitate survival (Shivlata and Satyanarayana, 2015). Notably, unexplored extreme habitats often yield novel and rare actinomycete isolates with antimicrobial properties (Aly et al., 2019). However, variations in environmental factors, isolation techniques, and screening methodologies significantly influence the diversity of isolated actinomycetes and the antimicrobial activity they exhibit.

Table 4 summarizes the diversity and antimicrobial activity of actinomycetes from extremophilic environments in Saudi Arabia.

**Table 4:** Diversity and antimicrobial activity of actinomycetes from extremophilic environments in Saudi Arabia.

Specimen type	Isolation site/ Study area	Isolated strains	Antimicrobial activity	Methods used to assess activity	Test microorganisms	Bioactive/ Chemical compound (s)	Reference
Deserts							
Soils	Saudi Arabia	*Thermomonospora* sp. T-SA-125	Antibacterial	Serial dilution agar method	*-Bacillus* spp. *-Staphylococcus aureus* *-Streptococcus faecalis* *-Lactobacillus* spp. *-Escherichia coli* *-Salmonella* spp. *-Klebsiella pneumoniae* *-Pseudomonas aeruginosa* *-Proteus* spp. *-Candida albicans* *-Trichophyton* spp. *-Microsporum* spp.	White powder antibiotic	Dewedar et al. (1979)
Soils	Jeddah & Baljurashi	*Streptomyces* spp.	Antibacterial	Agar-well diffusion method	*-Staphylococcus aureus* *-Staphylococcus epidermidis* *-Bacillus subtilis*	Crude extract	Alghamdi et al. (2021)
Soils	Al-Khurmah	*Streptomyces torulosus*, KH-4.	Antibacterial Antifungal	Agar well method	*-Staphylococcus aureus* *-Bacillus subtilis* *-Bacillus pumilus* *-Micrococcus luteus* *-Escherichia coli* *-Klebsiella pneumoniae* *-Pseudomonas aeruginosa* *-Candida albicans* *-Saccharomyces cerevisiae* *-Aspergillus flavus* *-Aspergillus fumigatous* *-Fusarium oxysporum*	Culture filtrate	Atta et al. (2010)
Soils	Thumamah, Kharj, Madina & Taif	*Streptomyces* spp.	Antibacterial Antifungal	Well diffusion method	*-Staphylococcus aureus* *-Bacillus subtilis* *-Shigella sonnei* *-Escherichia coli* *-Pseudomonas aeruginosa* *-Salmonella suis* *-Candida albicans*	-2,3-Butanediol -Octadecanal -Cyclobutanol -Oleic acid -Oleyl alcohol –Eicosanoic acid -Erucic acid -Glycerin -Heptanal -Acetic acid	Shinwari et al. (2013)
Soils	Riyadh province (Al-Qarinah, Al-Rabeya, Dahyat Namar, Hazlullah, Al-Muzahmiyah Al-Uraija, Ad Diriyah, Thumamah, Janadriyah, Al-Kharj)	Actinomycetes isolates	Antibacterial Antifungal	Cross streak method	*-Klebsiella pneumoniae* *-Escherichia coli* *-Proteus vulgaris* *-Pseudomonas aeruginosa* *-Staphylococcus aureus* *-Enterococcus faecalis* *-Candida albicans* *-Saccharomyces cerevisiae* *-Cryptococcus neoformans*	NA	Nithya et al. (2015)
Soils	Riyadh Province	*Streptomyces* sp. DA3-7	Antibacterial Antifungal	Cross streak plate, well-diffusion and disc diffusion methods	*-Klebsiella pneumoniae* *-Escherichia coli* *-Proteus vulgaris* *-Salmonella typhimurium* *-Pseudomonas aeruginosa* *-Staphylococcus aureus* *-Enterococcus faecalis* *-Candida albicans* *-Cryptococcus neoformans* *-Saccharomyces cerevisiae*	Pyridine-2,5-diacetamide	Nithya et al. (2018)
Soils	Makkah Province (Sayl Road, Makkah, Jeddah, Thuwal)	*-Streptomyces spiralis* *-Streptomyces spinoverrucosus* *-Streptomyces fimbriatus* *-Streptomyces carpinensis* *-Streptomyces geysiriensis* *-Streptomyces djakartensis* *-Streptomyces levis* *-Streptomyces atacamensis*	Antibacterial Antifungal	Cross Streak method	*-Streptococcus pneumoniae* -MRSA *-Staphylococcus aureus* *-Streptococcus pyogenes* *-Aspergillus niger* *-Candida albicans*	NA	Hausawi et al. (2021)
Soils	Al-Jouf province (Dumat Al-Jandal Qasr Kaff Ain Hawas Regions)	*Streptomyces* species	Antibacterial Antiprotozoal	Disc diffusion methods (Antibacterial) IC50 value (Antiprotozoal)	*-Streptococcus pneumoniae* *-Staphylococcus aureus* *-Escherichia coli* *-Bacillus cereus* *-Enterococcus faecalis* *-Salmonella* *typhimurium* *-Pseudomonas aeruginosa* *-Trypanosoma cruzi*	Flavonoids, phenolics, tocopherols & carotenoids	Almuhayawi et al. (2021)
Soils	Riyadh (Al Thumamah area)	*Streptomyces* species	Antibacterial Antifungal	Agar well diffusion method	*-Pseudomonas aeruginosa* *-Escherichia coli* *-Salmonella suis* *-Salmonella sonnei* *-Bacillus subtilis* *-Staphylococcus aureus* *-Candida albicans*	- 2,3-Butanediol, [R- (R*, R*)]- - Cyclobutanol - Octadecanal -Oleyl alcohol -Oleic acid -Eicosanoic acid -Acetic acid -Heptanal -Glycerin -Erucic acid	Alkhelaiwi et al. (2021)
Desert soils & medicinal plants	Riyadh (Thumama Desert)	*-Streptomyces paromomycinus* *-Streptomyces caelestis* *-Streptomyces lycii* *-Streptomyces cellulosae* *-Streptomyces roseicoloratus* *-Streptomyces albidochromogenes* *-Agromyces bauzanensis,* *-Lentzea* *-kentuckyensis,* *-Rhodococcus gordoniae,* *-Actinophytocola algeriensis,* *-Aeromicrobium massiliense,* *-Bacillus siamensis.*	Antibacterial Antifungal	Agar Well diffusion method	*-Klebsiella pneumoniae* *-Bacillus subtilis* -MRSA *-Candida albicans*	Essential and non-essential amino acids, valine, proline, ornithine, leucine. Hexadecatrienoic, hexadecanoic phenolics, flavonoids, beta- delta- and gama-tocopherol	Al-Khamash et al. (2023)
Caves							
Mine Soil	Madinah	Actinomycetes isolates (D 60)	Antibacterial	Agar well diffusion method	*-Staphylococcus aureus* *-Escherichia coli* *-Salmonella typhimurium* *-Aspergillus niger* *-Aspergillus flavus*	Culture filtrate	Al-Garni et al. (2014)
Soil	Riyadh (Hotel and Reda caves of Al Saman region)	*Streptomyces* spp. (*Streptomyces griseorubens* CL2)	Antibacterial	Agar plugs method	*-Staphylococcus aureus* -MRSA *-Enterococcus faecalis* *-Acinetobacter baumannii* *-Klebsiella oxytoca* *-Escherichia coli*	NA	Aly et al. (2020)
Soil	Saudi Arabia	*-Streptomyces* CL24 *-Amycolatopsis*	Antibacterial	Agar plug and Agar well diffusion methods	*-Staphylococcus aureus* -MRSA *-Enterococcus faecalis* *-Acinetobacter baumannii* *-Klebsiella oxytoca,* *-Escherichia coli*	Crude extract	Bahamdain (2020)
Soil	Al-Madinah (Umm Jirsan cave in Harrat Khyber)	*Streptomyces* spp. (*Streptomyces* sp. SAG-85)	Antibacterial	Cross streak, Agar plug and Agar well diffusion methods	*-Escherichia coli* *-Staphylococcus aureus* *-Klebsiella pneumoniae* *-Pseudomonas aeruginosa* *-Enterococcus faecalis* *Serratia marcescens -Salmonella* sp.	Crude extract	Al-Ghamdi et al. (2021)
Hot springs							
Sediments	Aseer region (Hot spring of Tharban)	*Streptomyces* sp. Al-Dhabi-1	Antibacterial Antifungal	Streaked perpendicular and agar well diffusion methods	*-Bacillus cereus* *-Escherichia coli* *-Enterococcus faecalis* *-Klebsiella pneumoniae* *-Proteus vulgaris* *-Staphylococcus epidermidis* *-Salmonella typhimurium* *-Staphylococcus aureus* *-Pseudomonas aeruginosa* *-Streptococcus agalactiae* *-Cryptococcus neoformans* *-Candida albicans*	-Benzeneacetic acid -Acetic acid 2-phenylethyl ester were the major compounds	Al-Dhabi et al. (2016)
Sediments	Aseer region (Hot spring of Tharban)	*Streptomyces* sp. Al-Dhabi-2	Antibacterial Antifungal	Cross-streak and agar well diffusion methods	*-Escherichia coli* *-Bacillus cereus* *-Klebsiella pneumoniae* *-Staphylococcus epidermidis* *-Enterococcus faecalis* *-Proteus vulgaris* *-Salmonella typhimurium* *-Staphylococcus aureus* *-Streptococcus agalactiae* *-Pseudomonas aeruginosa* *-Candida albicans* *-Cryptococcus neoformans*	The acetic acid, 2-phenylethyl ester, benzene acetic acid, acetic acid, 3,6-bis(2-methylpropyl)-, acetic acid methoxy-2-phenylethyl ester and diisopropyl ether were the major compounds	Al-Dhabi et al. (2019a, 2019b)
Mountains							
Soil	Aseer province (Riverbed of Rijal Alma)	Actinomycetes species	Antibacterial	Agar well diffusion method	*Staphylococcus aureus* *Streptococcus pyogenes* *Bacillus subtilis* *Escherichia coli* *Pseudomonas aeruginosa* *Klebsiella pneumoniae*	1,8,15,22-tetraaza-2,7,16,21-cyclooctacosane tetrone Cyclononasiloxane octadecamethyl Lycoxanthin Perylo[1,12-def]-1,3-dioxepin-5,11-dione,6,12-dihydroxy-8,9-bis(2- hydroxypropyl)-7,10-dimethoxy Ethyl iso-allocholate Octadecane, 3-ethyl-5-(2-ethylbutyl) Dasycarpidan-1-methanol acetate(ester) Hexadecanoic acid, 2, 3- dihydroxy propyl ester Propanoic acid, 2-(3-acetoxy-4,4,14-trimethylandrost-8-en-17-yl)- Heptadecane, 9-hexyl- Heptacosane Phthalic acid, butyl undecyl ester	Alqahtani et al. (2022)
Soil	Madinah (Uhud Mountain)	*Streptomyces macrosporus* OR916389 (MA-1) *Streptomyces rameus* OR916416 (MA-2)	Antibacterial Antifungal	Cross streak and agar well diffusion methods	*-Staphylococcus aureus* *-Bacillus cereus* *-Pseudomonas aeruginosa* *-Aspergillus niger* *-Penicillium* sp. *-Alternaria alternata* *-Fusarium solai.*	Culture filtrate	Albasri et al. (2024)
Mangroves							
Sediments	Jubail	*Streptomyces smyrnaeus* UKAQ_23	Antibacterial	Spot inoculation, disc diffusion, well-diffusion and micro broth dilution methods	*-Staphylococcus aureus* -MRSA *-Staphylococcus saprophyticus* *-Staphylococcus epidermidis* *-Streptococcus pyogenes* *-Streptococcus pneumoniae* *-Enterococcus faecalis* *-Bacillus cereus* *-Escherichia coli* *-Klebsiella pneumoniae* *-Pseudomonas aeruginosa* *-Proteus mirabilis* *-Proteus vulgaris* *-Salmonella typhimurium* *-Shigella flexneri* *-Candida albicans* *-Aspergillus niger*	Actinomycin X_2_ and Actinomycin D	Qureshi et al. (2021)

NA: Not Available IC_50_: Half maximal inhibitory concentration MRSA: Methicillin-resistant *Staphylococcus aureus*.

#### 4.3.1 Deserts

Desert ecosystems, characterized by extreme temperatures, low humidity, and scarce nutrient availability, represent an untapped source of novel microbial species and bioactive compounds. Actinomycetes in desert environments have demonstrated significant antimicrobial potential, particularly in producing antibiotics against multidrug-resistant pathogens. The methodology used for their isolation can significantly impact the recovery of bioactive strains. Pre-treatment techniques, such as heat treatment, dry-air exposure, and chemical enrichment using calcium carbonate, can selectively enrich for specific actinomycete populations while inhibiting the growth of competing microbes. Heat treatment, for example, eliminates non-spore-forming bacteria, thereby enhancing the isolation of thermophilic actinomycetes (Subramani and Aalbersberg, 2013). The choice of culture media also plays a crucial role in the metabolic expression of actinomycetes, as nutrient-rich media may favor fast-growing species, potentially overlooking slow-growing or rare actinomycetes (Nithya et al., 2015; Atta et al., 2010). Starch casein agar is widely used for isolating *Streptomyces* species due to its ability to support sporulation, whereas minimal media can promote the production of stress-induced metabolites not typically expressed in nutrient-rich conditions. Temperature fluctuations in deserts also induce physiological adaptations that enhance secondary metabolite production, often leading to bioactive compounds with unique structures (Almuhayawi et al., 2021).

Studies from various desert regions have underscored the diversity and antimicrobial potential of actinomycetes. In Egypt, Hozzein et al. (2011) identified diverse actinomycetes with notable antimicrobial activity. Ziyat et al. (2019) found that approximately 50% of actinomycetes isolated from Kazakhstan’s deserts exhibited antibacterial efficacy. In Pakistan, Fatima et al. (2019) isolated 110 actinomycete strains, with several demonstrating strong activity against MRSA. Similarly, actinomycetes isolated from Iran’s arid soils showed significant antimicrobial activity (Majidzadeh et al., 2021). Saleem et al. (2023) reported that actinomycetes from China’s Kubuqi Desert exhibited antimicrobial activity against multiple bacterial and fungal pathogens such as *S. aureus*, *Micrococcus luteus* (*M. luteus*), *B. subtilis*, *E. coli*, *Salmonella enterica* (*S. enterica*), and *Saccharomyces cerevisiae* (*S. cerevisiae*).

Despite these findings, research on actinomycetes from Saudi Arabia’s desert habitats—covering around 95% of the country—remains limited. The Rub’ al Khali (Empty Quarter) and An Nafud Desert, among the hottest and driest locations on earth, present unique conditions for microbial adaptation. Actinomycetes isolated from these regions demonstrate remarkable thermotolerance and antimicrobial potential that could address antibiotic resistance and meet the demand for new antimicrobial agents (Alotaibi et al., 2020; Almuhayawi et al., 2021; Alzahrani, 2021). For instance, a thermophilic strain, *Thermomonospora* spp. T-SA-125, obtained from desert soil in Saudi Arabia, produced a water-soluble antibiotic potent against Gram-positive as well as Gram-negative bacteria (Dewedar et al., 1979). *Streptomyces jeddahensis* sp. nov., isolated near Jeddah, withstands temperatures up to 50°C (Röttig et al., 2017). Alghamdi et al. (2021) identified actinomycetes from Jeddah and Baljurashi soil samples, with some closely related to *Streptomyces* species showing antibacterial activity, specifically against *S. aureus*, *Staphylococcus epidermidis* (*S. epidermidis*), and *B. subtilis*. Atta et al. (2010) identified *Streptomyces torulosus* KH-4 from the Al-Khurmah desert and as notably effective against both Gram-positive and Gram-negative bacteria, as well as fungi. In a similar manner, Shinwari et al. (2013) identified *Streptomyces* species, isolated from soils of the Saudi desert. Most of these isolates exhibited significant antimicrobial activity against pathogens such as *S. aureus*, *B. subtilis*, *E. coli*, *P. aeruginosa*, *Salmonella suis*, *S. sonnei*, and *C. albicans*. In another study, Nithya et al. (2018) revealed that a crude extract from *Streptomyces* sp. DA3-7 obtained from a desert in Riyadh had antibacterial and cytotoxic properties.

Recent Saudi research continues to uncover promising desert-derived actinomycetes. Alkhelaiwi et al. (2021) isolated 30 actinomycetes strains (F1-30) from soil samples obtained from the Al Thumamah area of Saudi Arabia. Ten strains exhibited antimicrobial activity against at least one pathogen. Almuhayawi et al. (2021) isolated 21 actinomycetes from the Al-Jouf desert in Saudi Arabia. The isolates, mostly *Streptomyces* species, generated a substantial quantity of bioactive compounds exhibiting notable antioxidant, antibacterial, and antiprotozoal activities against *T. cruzi*. Desert plant associations impact actinomycetes communities. Root-associated actinomycetes exhibit increased antimicrobial activity due to plant-microbe interactions. Hausawi et al. (2021) discovered *Streptomyces* species from desert soil surrounding various plants in Makkah, Saudi Arabia. These isolates exhibited activity against pathogens including *Streptococcus pneumoniae* (*S. pneumoniae*), MRSA, *S. aureus*, *S. pyogenes, A. niger,* and *C. albicans*, indicating their potential as novel antibiotic sources for medicinal use. Additionally, Al-Khamash et al. (2023) found that several plant-associated actinomycetes strains, collected from the Al Thumama desert in the Riyadh region of Saudi Arabia, showed antimicrobial activity against *K. pneumoniae*, *B. subtilis*, and *C. albicans*.

Desert-derived actinomycetes represent an emerging frontier in microbial biotechnology. While Saudi Arabia’s deserts hold immense potential for novel antibiotic discovery, variations in isolation techniques and environmental factors significantly impact microbial diversity and bioactivity. Addressing these variables through standardized methodologies and advanced genomic approaches to reveal biosynthetic gene clusters responsible for antibiotic production will pave the way for more consistent and impactful discoveries.

#### 4.3.2 Caves

Cave environments hold great potential for discovering new antimicrobial compounds due to the intense competition among microorganisms for limited nutrients. This competition drives the production of antimicrobial substances as a survival mechanism. For example, *Streptomyces* isolated from volcanic caves has been shown to produce high levels of secondary metabolites. Unique cave conditions—such as reduced light, high humidity, low nutrient availability, high concentration of minerals (carbonate, sulphates, phosphates, and potassium), acidity, and lack of oxygen— enhance microbial production of these compounds (Jones, 2001; Cheeptham, 2012; Farda et al., 2022). Recent years have witnessed an increasing interest in the examination of microbial diversity in caves owing to their harsh environments (Groth et al., 2002; Duangmal et al., 2012). Actinomycetes, prevalent in these ecosystems, are particularly attractive sources for novel drug discoveries, demonstrating potential for pharmaceutical applications (Rangseekaew and Pathom-Aree, 2019; Farda et al., 2022). Yücel and Yamaç (2010) reported that *Streptomyces* sp. 1,492, isolated from Turkish karstic caves, exhibited antimicrobial activity against multidrug-resistant bacteria such as MRSA and *A. baumannii*. Similarly, *Streptomyces badius* (S142) and *Actinoplanes friuliensis* (S761) isolated from soil, rock-soil, and bat manure of Shuanghe Cave in China were found to inhibit various pathogenic microorganisms (Long et al., 2019). A comprehensive study in Hampoeil Cave, Iran, identified 76 actinobacterial strains, including genera such as *Streptomyces, Micromonospora, Micrococcus, Kocuria,* and *Corynebacterium*. Of these, 25.3% exhibited antimicrobial activity (Hamedi et al., 2019). Similarly, Iquebal et al. (2021) isolated 235 bacterial strains from Pukzing Cave, India, with actinomycetes representing the predominant bacterial group. Approximately 20.4% of these isolates displayed broad-spectrum antimicrobial activity against pathogens like *S. aureus*, *P. aeruginosa*, *E. coli*, *M. luteus*, *B. subtilis*, and *C. albicans*.

Saudi Arabia is known for its rich geological landscape, including some of the rarest wild caves with remarkable geological and geomorphic features. However, the microbial content of these caves has been largely understudied. Few geological or microbial investigations have been conducted in the caves of Saudi Arabia, leaving a significant knowledge gap. Caves such as Jabal Al Qarah, Harrat Khaybar lava caves Dahl Rumahah, Kahf Al Shuwaymis, and the Umm Jirsan Lava-Tube are among the oldest known caves explored for their ecological and microbial diversity (Pint, 2009; Jastaniah et al., 2019a). These caves offer an uncharted environment for the discovery of extremophilic bacteria, particularly actinomycetes, which exhibit remarkable adaptations to oligotrophic conditions, low temperatures, and high humidity levels. Notably, actinomycetes isolated from Saudi caves, such as those in Jabal Al-Qarah, have demonstrated the ability to thrive in nutrient-deficient environments while exhibiting resilience to extreme physicochemical conditions. Caves rich in minerals such as sulfur, limestone, and gypsum serve as specialized habitats for mineral-tolerant actinomycetes, many of which possess bioactive properties. These microorganisms are of particular interest due to their potential to produce antimicrobial compounds and other secondary metabolites with biotechnological applications (Jastaniah et al., 2019a; Aly et al., 2020; Al-Ghamdi et al., 2021).

Initial microbial investigations have begun to characterize Saudi cave microbiomes. Amasha (2018) reported diverse microbial taxa, including *Proteobacteria, Actinobacteria, Firmicutes,* and *Bacteroidetes*, from Mossy, Hotel, and Reda Caves in the Summan region near Riyadh. Earlier work in Ghar Al Hibashi Cave, southeast of Makkah, revealed a diverse microbial assemblage, underscoring the potential of Saudi caves as reservoirs for novel bioactive compounds (Amasha, 2012). More recent studies have identified promising actinomycetes isolates from Saudi caves. Aly et al. (2020) and Bahamdain (2020) isolated *Streptomyces griseorubens* CL2 and *Streptomyces* CL24 from a cave in the Saman region, demonstrating potent activity against MRSA and *E. faecalis*. Al-Ghamdi et al. (2021) investigated the Umm Jirsan lava tube in Harrat Khaybar, isolating actinomycetes with strong antimicrobial properties against MRSA, *S. aureus*, *E. faecalis, Serratia marcescens* (*S. marcescens*), *Salmonella* sp., *E. coli*, *P. aeruginosa* and *K. pneumoniae.* Extreme cave environments, characterized by nutrient limitations and high humidity, support specialized microbial communities that produce antimicrobials with significant potential for agricultural, industrial, and medical applications. While global research has identified numerous novel strains, Saudi Arabian caves remain an underexplored resource. Expanding research in these ecosystems through standardized methodologies, genomic approaches, and interdisciplinary collaborations could unlock unique microbial taxa for biotechnological advancements.

#### 4.3.3 Hot springs

Hot springs, characterized by elevated temperatures and unique physicochemical conditions, provide an ideal habitat for hyperthermophilic microorganisms. These microbes, thriving at temperatures between 45°C and 80°C, possess specialized membrane lipids rich in saturated, straight-chain fatty acids that ensure stability and fluidity under extreme conditions (Cherifa et al., 2023). Their metabolic adaptations make them valuable for biotechnological applications.

Microbial communities in hot springs encompass diverse thermophiles, including sporulating and non-sporulating bacilli, actinomycetes, and cyanobacteria (Jiang and Xu, 1993). Among them, actinomycetes—especially *Streptomyces* spp.—exhibit remarkable potential for synthesizing antimicrobial compounds and industrially relevant enzymes (Bahamdain et al., 2020; Cherifa et al., 2023). These compounds have wide-ranging uses, spanning from pharmaceutical development to bioremediation. However, the isolation and retention of thermophilic actinomycetes in pure cultures, despite their potential, continue to pose significant challenges. This has constrained research opportunities and impeded the identification of novel substances from these organisms (Alrumman et al., 2019; Ruwandeepika et al., 2022; Albasri et al., 2024).

Despite increasing interest in the thermophilic secondary metabolism, Saudi Arabia’s hot springs remain relatively unexplored. With approximately ten geothermal springs in regions such as Jizan, Al-Lith and Aseer, these sites present varied thermal and chemical conditions that support extremophiles (Khiyami et al., 2012; El-Gayar et al., 2017; Alrumman et al., 2018; Yasir et al., 2019). Several studies have reported actinomycetes from these environments capable of producing heat-stable enzymes and antimicrobial compounds, making them promising candidates for pharmaceutical and industrial applications (Al-Dhabi et al., 2019a; AlSediy et al., 2022).

Significant findings include the isolation of *Streptomyces Al-Dhabi-1* from the Tharban hot spring, which exhibits potent antibacterial activity against *Streptococcus agalactiae* (*S. agalactiae*) and *K. pneumoniae*, along with antifungal properties against *Cryptococcus neoformans* (*C. neoformans*), *C. albicans*, *Trichophyton mentagrophytes* (*T. mentagrophytes*), and *A. niger* (Al-Dhabi et al., 2016). Another strain, *Streptomyces* sp. *Al-Dhabi − 2*, demonstrated broad-spectrum antimicrobial activity against *B. cereus, E. faecalis, S. typhimurium*, and *P. aeruginosa* (Al-Dhabi et al., 2019a). Further studies by Bahamdain et al. (2020) identified *Streptomyces* strains from soil sediments, hot spring water, and inner channel walls of the Oyun Al-Haar Al-Lith hot spring, which produce key enzymes such as keratinase, gelatinase, chitinase, lipase, amylase, and protease, with applications in pharmaceuticals, biofuel production, and waste degradation.

Recent investigations into microbial diversity in soils from hot springs in Jizan, Al-Lith, and Tabuk have revealed a diverse range of dominant microbial phyla, including *Acidobacteriota*, *Actinobacteriota*, *Desulfobacterota*, *Firmicutes*, *Halanaerobiaeota*, *Nitrospirota*, and *Thermotogota*. Notably, *Actinobacteriota* were most prevalent in Al-Lith, while *Deinococcota* dominated Jizan and *Chloroflexi* in Tabuk. While traditional culture-based methods have identified key thermophilic actinomycetes, they may underestimate the overall microbial diversity. Advanced molecular techniques, such as metagenomics and 16S rRNA sequencing, are increasingly being utilized to characterize microbial populations in extreme environments, providing a more comprehensive understanding of their diversity and ecological roles (AlSediy et al., 2022).

These findings suggest that Saudi Arabia’s hot springs harbor diverse thermophilic actinomycetes with significant antimicrobial and industrial potential. However, further genomic, biochemical, and ecological studies are required to fully harness their capabilities. Integrating modern omics technologies, including metabolomics, transcriptomics, and genomics, will facilitate the discovery of novel bioactive compounds, elucidate gene functions, metabolic pathways, and biosynthetic potential. Such advancements will enhance the discovery of new antimicrobial agents and industrially valuable enzymes, reinforcing the importance of these unique environments in biotechnology.

#### 4.3.4 Mountains

Mountainous regions, characterized by diverse heights, temperature fluctuations, and various soil compositions, offer a distinctive environment for microbial communities, especially actinomycetes. Adverse environmental conditions, like cold temperatures, elevated UV radiation, and restricted nutrition availability, serve as selective pressures, driving the evolution of actinomycetes strains with enhanced survival mechanisms. These adaptations often include the production of bioactive compounds with potent antimicrobial properties, which help the organisms compete with other microorganisms in their habitat. As a result, actinomycetes isolated from mountain soils have shown considerable potential in the development of new antibiotics, especially at a time when antimicrobial resistance is a growing global concern (Bull, 2011; Shivlata and Satyanarayana, 2015).

Studies across various mountainous regions have revealed the vast antimicrobial potential of actinomycetes. Gurung et al. (2009) isolated 79 actinomycetes from the Kalapatthar region of Mount Everest, with many demonstrating broad-spectrum antibacterial activity, including against MDR pathogens such as MRSA. Similarly, Zhu et al. (2014) examined over 800 actinomycetes from the Qinling and Himalayan mountains in China, identifying 96 isolates—primarily from *Streptomyces*—capable of inhibiting ESKAPE pathogens (*E. faecium*, *S. aureus*, *K. pneumoniae*, *A. baumannii*, *P. aeruginosa*, and *Enterobacter cloacae*), which are known for their resistance to conventional treatments. In Yemen’s Hadhramout governorate, Bawazir et al. (2017) identified *Nocardia otitidiscaviarum*, a potent isolate with broad-spectrum antibacterial activity. These findings collectively underscore the untapped potential of mountain-derived actinomycetes in novel antibiotic discovery.

The mountainous regions of Saudi Arabia, including the Aseer and Hijaz ranges, offer unique cooler, and more humid microclimates that contribute to the country’s environmental diversity. These conditions make the region promising for the exploration of bioactive microorganisms. Recent studies have highlighted the antimicrobial potential of actinomycetes from these regions. Alqahtani et al. (2022) studied actinomycetes isolated from the riverbed of Rijal Alma in Aseer, revealing strong antibacterial activity, particularly against *E. coli* and *B. subtilis*. Expanding on this, Albasri et al. (2024) focused on thermophilic actinomycetes from Uhud Mountain in Madinah, isolating strains closely related to *Streptomyces macrosporus* and *Streptomyces rameus*. These isolates exhibited significant antibacterial activity against *B. cereus* and *S. aureus* and notable antifungal activity against *A. niger*, *Alternaria alternata* (*A. alternata*), and *Penicillium* species. The discovery of such promising isolates in Saudi Arabia underscores the region’s potential as a reservoir of novel actinomycetes with pharmaceutical significance.

#### 4.3.5 Mangroves

Mangroves are unique coastal ecosystems located in the intertidal zones of tropical and subtropical regions, particularly in Southeast Asia. These environments are characterized by extreme conditions such as high salinity, tidal fluctuations, and temperature variations, yet they support diverse microbial communities, including actinomycetes. The ability of actinomycetes to thrive in such harsh conditions suggests that they possess unique metabolic pathways, some of which may contribute to the production of bioactive compounds with potential applications in medicine and industry (Law et al., 2019).

Actinomycetes, particularly *Streptomyces* species, are prominent soil inhabitants within mangrove ecosystems and have gained considerable attention due to their capacity to produce a wide array of bioactive compounds, including antibiotics, antifungals, anticancer agents, and antioxidants. Studies conducted across diverse mangrove habitats have demonstrated significant variations in actinomycetes diversity and bioactivity, often influenced by geographical and environmental factors (Naikpatil and Rathod, 2011; Rajkumar et al., 2012; Ara et al., 2013). Many studies across East, South, and Southeast Asia have highlighted the diversity of mangrove actinomycetes as a rich source of novel bioactive compounds, many of which demonstrate strong antagonistic activity against a wide spectrum of microorganisms, including multidrug-resistant pathogens (Satheeja and Jebakumar, 2011; Sathiyaseelan and Stella, 2011; Govindarajan et al., 2021).

India possesses one of the most extensive mangrove ecosystems in South Asia, and numerous researchers have investigated the diversity of actinomycetes inside these areas. For example, Mitra et al. (2008) studied the distribution of actinomycetes in the Sundarbans mangroves and identified that the actinomycetes obtained from soil samples were classified into nine genera, displaying significant antimicrobial potential. Subsequent research in the Sundarbans by Arifuzzaman et al. (2010) and Sengupta et al. (2015) reinforced these findings, confirming the antimicrobial efficacy of various isolates. Other Indian mangroves, such as those in the Bay of Bengal and Pichavaram, Tamil Nadu, have also been explored for their actinobacterial potential. Ramesh and Mathivanan (2009) found that isolates from the Bay of Bengal exhibited strong antimicrobial activity against human pathogens, including *P. aeruginosa*, *E. coli*, *B. subtilis*, *S. epidermidis*, and *C. albicans.* Similarly, Usha et al. (2010) identified *Streptomyces parvulus* KUAP106 from Pichavaram mangroves, demonstrating potent antibacterial effects against *B. subtilis*, *S. aureus*, *P. aeruginosa*, and *E. coli*. Additional studies by Mangamuri et al. (2012) and Mangamuri et al. (2014) evaluated actinomycetes from the Nizampatnam and Coringa mangroves, which exhibited antimicrobial effects against *E. coli*, *S. aureus*, and *C. albicans*.

Mangroves in Southeast Asia exhibit comparable diversity of actinomycetes. In Malaysia, Lee et al. (2014) isolated multiple strains with significant antibacterial properties against a range of pathogens, including *B. subtilis*, *B. cereus*, *E. faecalis*, *S. epidermidis*, MRSA, *S. typhi*, *Acinetobacter calcoaceticus*, and *Aeromonas hydrophila*. Studies in Indonesia (Retnowati et al., 2017) reported actinobacteria from genera such as *Streptomyces*, *Amycolatopsis*, *Nocardiopsis*, and *Saccharomonospora*, highlighting their biotechnological potential. In East Asia, Hong et al. (2009) documented over 2,000 actinomycete isolates from Chinese mangroves, predominantly *Streptomyces*, with notable antimicrobial activity against *C. albicans* and *S. aureus*. Wei et al. (2010) further identified antibiotic-producing strains from *Streptomyces*, *Micromonospora*, and *Nocardia*, suggesting their relevance in infection treatment.

Research on Saudi Arabian mangroves, particularly those in Jazan and Jubail, is gaining momentum, revealing their potential as sources of novel actinomycetes (Reyad, 2013; Qureshi et al., 2021). Isolates from these environments have demonstrated promising antimicrobial activity against multidrug-resistant pathogens such as MRSA and have exhibited capabilities in producing industrially significant hydrolytic enzymes like amylase, cellulase, and lipase. These findings emphasize the untapped potential of arid-region mangroves in pharmaceutical (e.g., antibiotics and anticancer) and industrial biotechnology (e.g., biofuel production, bioremediation. and food industries). Furthermore, sustainability should remain a central focus to ensure long-term benefits without compromising mangrove conservation.

### 4.4 Other unique environments

Actinomycetes are widely distributed across various ecosystems, extending beyond soil and marine environments to unique niches. These include the digestive tracts of arthropods, airborne spores, endophytic relationships with plants, and organic waste environments such as composts.

#### 4.4.1 Actinomycetes in animal-associated niches

Actinomycetes are found in the digestive tracts of various arthropods, where they play crucial roles in host defense and microbiome stability (Mitousis et al., 2020; Olano and Rodríguez, 2024). For instance, African *Tetraponera penzigi* ants harbor *Streptomyces formicae*, which produces potent antibiotics known as formicamycins. These compounds exhibit significant inhibitory effects against multidrug-resistant pathogens such as MRSA and VRE (Qin et al., 2017). Additionally, rare actinomycetes, such as *Nocardiosis* sp., have been identified in the gut of bees and characterized by the expression of antibiotic biosynthetic genes (Selim et al., 2021). In Saudi Arabia, *Streptomyces* species were isolated from the gut of *Apis mellifera yemintica* honeybees, with extracts demonstrating significant antibacterial, antifungal, and anticancer properties (Al-Enazi et al., 2022). Similarly, earthworm castings serve as reservoirs of actinomycetes with potential antimicrobial activity and industrial enzyme production. Dominant genera in these castings include *Streptomyces* and *Streptosporangium*. Notably, *Streptomyces* isolates from earthworm castings exhibit antagonistic activity against wood-degrading fungi, suggesting applications in veterinary and human medicine (Selim et al., 2021).

#### 4.4.2 Airborne and atmospheric Actinomycetes

Actinomycetes spores are pervasive in the atmosphere, where they contribute to respiratory allergies and have been identified as producers of bioactive compounds. Airborne *Nocardia* spores, for example have been associated with antimicrobial production (Lacey and Crook, 1988; Selim et al., 2021). However, further studies are needed to assess their impact on respiratory health and their potential industrial applications in bioprospecting novel antibiotics.

#### 4.4.3 Plant-associated and endophytic Actinomycetes

There are many reports demonstrating the distribution of endophytic actinomycetes in diverse plants, such as crop plants, medicinal plants, halophytes, and some woody tree species, mainly in the roots, stems or leaves of these plants. They are considered useful microorganisms due to their roles as a defense barrier against phytopathogens as well as their application in medical and industrial fields (Aly et al., 2019; Selim et al., 2021; Alzahrani et al., 2022). For example, *Streptomyces parvulus* Av-R5, associated with the root of *Aloe vera* exhibited strong antibacterial activity against multidrug-resistant *S. aureus, K. pneumoniae*, and *A. niger* (Chandrakar and Gupta, 2019). These findings highlight the potential of endophytic actinomycetes in the production of novel bioactive compounds, which can be further explored for applications in agriculture and medicine.

#### 4.4.4 Compost and decay-associated Actinomycetes

Composts and decayed fodder represent another critical habitat for actinomycetes due to their conducive damp, aerobic, and alkaline environments. In these environments, thermophilic actinomycetes, such as *Saccharomonospora*, *Saccharopolyspora*, *Streptomyces*, *Thermoactinomyces*, *Thermobifida*, and *Thermomonospora*, play an essential role in organic matter degradation. These organisms frequently form surface blooms, colloquially known as “fire tooth,” as temperatures rise during composting processes (Dilip et al., 2013; Bawazir and Shantaram, 2018; Ngamcharungchit et al., 2023). Research in Saudi Arabia has underscored the adaptability of actinomycetes to extreme environmental conditions. Isolates obtained from poultry farm waste, soil, water, fodder, and feathers have demonstrated promising biotechnological applications, including organic waste degradation and contributions to environmental remediation (Aly and Tork, 2018; Khalel et al., 2020).

## 5 Challenges and future trends

Despite the remarkable antimicrobial potential of actinomycetes from diverse Saudi Arabian habitats, several challenges hinder their full exploitation for pharmaceutical and biotechnological applications. These challenges span across various stages, from strain isolation to industrial-scale production and regulatory approval.

### 5.1 Challenges

- *Limited Cultivability of Rare Actinomycetes*: Many actinomycetes, particularly those from extreme environments, remain uncultivable under standard laboratory conditions. Conventional methods often fail to replicate the biochemical and ecological conditions required for their growth. Addressing this challenge necessitates customized media, advanced isolation methodologies (e.g., pretreatment), and *in situ* cultivation techniques such as isolation chip (iChip) technology, which facilitates the growth of previously unculturable microorganisms by preserving their natural environmental conditions. Additionally, co-cultivation strategies that simulate microbial interactions can enhance the growth of certain actinomycetes by mimicking their ecological niches (Hug et al., 2018).
- *Complexity of Biosynthetic Gene Clusters (BGCs)*: The production of secondary metabolites is regulated by intricate BGCs, many of which remain silent under conventional culture conditions. Genome mining and activation strategies, such as co-cultivation and chemical elicitors, are needed to enhance metabolite production (van Bergeijk et al., 2020).
- *High Costs of Large-Scale Production*: The transition from laboratory-scale isolation to industrial-scale antibiotic production faces economic barriers, including costly fermentation processes and low yields of bioactive compounds (De Simeis and Serra, 2021).
- *Regulatory and Safety Challenges*: The approval of new antimicrobial agents requires extensive preclinical and clinical evaluations, which are time-consuming and costly (De Simeis and Serra, 2021; Jagannathan et al., 2021).
- *Environmental and Sustainability Concerns*: Overharvesting of natural habitats for actinomycete isolation may impact local ecosystems. Sustainable bioprospecting practices must be implemented to balance microbial conservation and discovery efforts (Jagannathan et al., 2021).
- *Limited Integration of Advanced Technologies*: A significant limitation in actinomycete research is the inadequate integration of modern technologies, including comprehensive genomic, metagenomic, and metabolomic characterization. The application of genome mining, synthetic biology, omics technologies (genomics, transcriptomics, proteomics, and metabolomics), AI-driven bioinformatics, and CRISPR-based gene editing remains underutilized, constraining the identification of novel BGCs responsible for antibiotic production (Ngamcharungchit et al., 2023).
- *Challenges in Scalable Cultivation Techniques*: While metagenomic-based screening approaches provide an alternative to direct cultivation by enabling the identification and characterization of novel microbial strains through environmental DNA analysis, their scalability remains a challenge. Further technological refinements are required to integrate these techniques into high-throughput screening pipelines (Ngamcharungchit et al., 2023).

### 5.2 Future trends and opportunities

- *Advancements in Metagenomics and Genome Mining*: Novel actinomycetes and their bioactive metabolites can be identified using metagenomic approaches, whole-genome sequencing, and bioinformatics tools, bypassing the need for traditional cultivation (Ngamcharungchit et al., 2023).
- *AI and Machine Learning in Drug Discovery:* AI-driven predictive models can streamline the discovery of novel antimicrobial compounds by analyzing large datasets of biosynthetic gene clusters and metabolic pathways (Ngamcharungchit et al., 2023)
- *CRISPR and Synthetic Biology Approaches*: Genetic engineering and synthetic biology can be utilized to enhance the biosynthetic potential of actinomycetes, leading to increased production of antibiotics and novel compounds (Mitousis et al., 2020).
- *Bioprocess Optimization for Industrial Applications*: The development of advanced fermentation techniques, such as continuous fermentation and metabolic engineering, can improve yield and reduce production costs (De Simeis and Serra, 2021).
- *Integration of Omics Technologies*: The “One Strain–Many Compounds” (OSMAC) approach, AI-driven drug discovery, bioinformatics, and CRISPR-based activation of cryptic BGCs offer promising avenues for unlocking novel bioactive compounds. Omics technologies provide comprehensive insights into gene expression, regulatory networks, and metabolite biosynthesis, thereby streamlining drug discovery and optimizing strain engineering (Ngamcharungchit et al., 2023).
- *Bioprocessing and Drug Delivery Innovations*: Bioprocessing advancements, such as scalable fermentation and nanotechnology-based drug delivery systems, can enhance the stability, efficacy, and bioavailability of actinomycete-derived compounds (Ngamcharungchit et al., 2023).
- *Expanding Bioprospecting to Novel Ecological Niches*: The exploration of extreme environments, including deserts, caves, marine ecosystems, and endophytic niches, is broadening the scope of actinomycete biodiversity and uncovering new antimicrobial metabolites. Expanding cultivation techniques and bioprospecting in these unique ecosystems will facilitate the discovery of actinomycetes with unique biosynthetic potential (Ngamcharungchit et al., 2023)
- *Non-Traditional Therapeutic Strategies Against Antibiotic Resistance*: In response to the rising threat of antimicrobial resistance, alternative treatment approaches are gaining attention. These include quorum sensing inhibitors, phage therapy, and anti-virulence drugs derived from actinomycetes to reduce resistance development (Jagannathan et al., 2021).
- *Application Beyond Antimicrobials*: Actinomycete-derived bioactive compounds are increasingly being explored for their potential applications beyond antibiotics, including oncology, immunotherapy, and agriculture. These compounds exhibit anticancer, anti-inflammatory, and immunomodulatory properties, opening new avenues for therapeutic development (Ngamcharungchit et al., 2023).
- *Advanced Analytical Techniques for Compound Characterization*: The identification and structural elucidation of novel bioactive compounds require cutting-edge analytical technologies, including mass spectrometry (MS), liquid chromatography (LC), gas chromatography (GC), and nuclear magnetic resonance (NMR) spectroscopy. These methods are essential for determining the molecular structures, biosynthetic pathways, and mechanisms of action of new antimicrobial compounds, thereby accelerating drug discovery and development (Hug et al., 2018; Jagannathan et al., 2021).
- *Interdisciplinary Collaborations*: Strengthening partnerships between microbiologists, biotechnologists, and pharmaceutical industries is crucial to accelerating the development of actinomycete-derived antibiotics (Hug et al., 2018).

## 6 Conclusion

This systematic review highlights the diverse actinomycete populations across Saudi Arabia’s terrestrial, aquatic, and extremophilic environments, emphasizing their untapped potential as sources of novel bioactive compounds. Actinomycetes from these unique ecosystems demonstrate significant antimicrobial efficacy against multidrug-resistant pathogens, reinforcing their importance in combating the global antimicrobial resistance crisis. However, challenges such as strain cultivability, biosynthetic gene activation, and industrial scalability must be addressed to fully harness their potential. Integrating advanced genomic, bioinformatics, and synthetic biology tools offers promising solutions to overcome these barriers. Future research should prioritize interdisciplinary collaborations, sustainable bioprospecting, and bioprocess optimization to facilitate the translation of actinomycete-derived bioactive compounds into clinically viable therapeutics. The unique microbial biodiversity of Saudi Arabia represents an invaluable resource for the discovery of next-generation antibiotics and other pharmaceutical innovations.

## Footnotes

^1^ https://lpsn.dsmz.de/phylum/actinomycetota

## Glossary

AMR: Antimicrobial resistance
WHO: World Health Organization
MDR: Multidrug-resistant
MRSA: Methicillin-resistant *Staphylococcus aureus*
VRSA: Vancomycin-resistant *Staphylococcus aureus*
VRE: Vancomycin-resistant *Enterococcus* spp
PRSP: Penicillin-resistant *Streptococcus pneumoniae*
ESBL: Extended-spectrum beta-lactamase
*E. coli*: *Escherichia coli*
*K. pneumoniae*: *Klebsiella pneumoniae*
CRE: Carbapenem-resistant Enterobacteriaceae
*P. aeruginosa*: *Pseudomonas aeruginosa*
*A. baumannii*: *Acinetobacter baumannii*
MDR-TB: Multidrug-resistant *Mycobacterium tuberculosis*
BGCs: Biosynthetic gene clusters
PKSs: Polyketide synthases
NRPSs: Non-ribosomal peptide synthetases
*S. aureus*: *Staphylococcus aureus*
*B. cereus*: *Bacillus cereus*
*S. typhi*: *Salmonella typhi*
*C. albicans*: *Candida albicans*
*A. niger*: *Aspergillus niger*
*A. flavus*: *Aspergillus flavus*
*S. rochei*: *Streptomyces rochei*
*B. subtilis*: *Bacillus subtilis*
*S. enteritidis*: *Salmonella enteritidis*
*A. fumigatus*: *Aspergillus fumigatus*
*F. solani*: *Fusarium solani*
*S. sonnei*: *Shigella sonnei*
*E. faecalis*: *Enterococcus faecalis*
*S. pyogenes*: *Streptococcus pyogenes*
MIC: Minimum inhibitory concentration
*T. cruzi*: *Trypanosoma cruzi*
*F. oxysporum*: *Fusarium oxysporum*
*S. typhimurium*: *Salmonella typhimurium*
*P. mirabilis*: *Proteus mirabilis*
*E. faecium*: *Enterococcus faecium*
*M. luteus*: *Micrococcus luteus*
*S. enterica*: *Salmonella enterica*
*S. cerevisiae*: *Saccharomyces cerevisiae*
*S. epidermidis*: *Staphylococcus epidermidis*
*S. pneumoniae*: *Streptococcus pneumoniae*
*S. marcescens*: *Serratia marcescens*
*S. agalactiae*: *Streptococcus agalactiae*
*C. neoformans*: *Cryptococcus neoformans*
*T. mentagrophytes*: *Trichophyton mentagrophytes*
*A. alternata*: *Alternaria alternata*
omics: genomics, transcriptomics, proteomics, and metabolomics
OSMAC: “One Strain–Many Compounds”
CRISPR: Clustered Regularly Interspaced Short Palindromic Repeats
MS: Mass Spectrometry
LC: Liquid Chromatography
GC: Gas Chromatography
NMR: Nuclear Magnetic Resonance spectroscopy
