# Correction: Exploring the diversity and antimicrobial potential of actinomycetes isolated from different environments in Saudi Arabia: a systematic review

**DOI:** 10.3389/fmicb.2025.1689464

**Published:** 2025-09-01

**Authors:** Noof Refat Helmi

**Affiliations:** Department of Clinical Microbiology and Immunology, Faculty of Medicine, King Abdulaziz University, Jeddah, Saudi Arabia

**Keywords:** actinomycetes, *Streptomyces*, antimicrobial activity, antimicrobial resistance, extreme environment, bioactive compound, Saudi Arabia

In the published article, the article type was erroneously categorized as “Review”.

The correct article type is “Systematic Review,” as the manuscript was prepared and structured in accordance with systematic review methodology, including comprehensive database search strategies, predefined inclusion and exclusion criteria, quality assessment of included studies, and adherence to PRISMA reporting guidelines.

The original version of this article has been updated.

